# Marine and Freshwater Feedstocks as a Precursor for Nitrogen-Containing Carbons: A Review

**DOI:** 10.3390/md16050142

**Published:** 2018-04-26

**Authors:** Anna Ilnicka, Jerzy P. Lukaszewicz

**Affiliations:** 1 Faculty of Chemistry, Nicolaus Copernicus University, Gagarina 7, 87-100 Torun, Poland; jerzy_lukaszewicz@o2.pl; 2 Centre for Modern Interdisciplinary Technologies, Nicolaus Copernicus University, Wilenska 4, 87-100 Torun, Poland

**Keywords:** marine feedstock processing, marine-derived products, nitrogen-doped carbon, marine animals, marine plants

## Abstract

Marine-derived as well as freshwater feedstock offers important benefits, such as abundance, morphological and structural variety, and the presence of multiple elements, including nitrogen and carbon. Therefore, these renewal resources may be useful for obtaining N- and C-containing materials that can be manufactured by various methods, such as pyrolysis and hydrothermal processes supported by means of chemical and physical activators. However, every synthesis concept relies on an efficient transfer of nitrogen and carbon from marine/freshwater feedstock to the final product. This paper reviews the advantages of marine feedstock over synthetic and natural but non-marine resources as precursors for the manufacturing of N-doped activated carbons. The manufacturing procedure influences some crucial properties of nitrogen-doped carbon materials, such as pore structure and the chemical composition of the surface. An extensive review is given on the relationship between carbon materials manufacturing from marine feedstock and the elemental content of nitrogen, together with a description of the chemical bonding of nitrogen atoms at the surface. N-doped carbons may serve as effective adsorbents for the removal of pollutants from the gas or liquid phase. Non-recognized areas of adsorption-based applications for nitrogen-doped carbons are presented, too. The paper proves that nitrogen-doped carbon materials belong to most of the prospective electrode materials for electrochemical energy conversion and storage technologies such as fuel cells, air–metal batteries, and supercapacitors, as well as for bioimaging. The reviewed material belongs to the widely understood field of marine biotechnology in relation to marine natural products.

## 1. Introduction

The aim of this paper is to review the advantages of marine/freshwater feedstock over synthetic and natural but non-marine resources as precursors for the manufacturing of nitrogen-doped activated carbons, together with the description of such carbon applicability. The subject may be related to the widely understood fields of marine biotechnology, microbiology, and/or the engineering of marine natural products. Nitrogen-doped carbon materials are in fact a result of synthesis based on marine natural products resources such as algae, fish, microorganisms, chitin, and chitosan.

Carbon is a unique chemical element occurring in nature in many diversified forms. Graphite, coal, carbon black, and diamond all are almost pure, natural forms of carbon. Artificial carbon-based materials can be produced in various forms, such as activated carbon, carbon nanotubes, fullerenes, or graphene. Numerous research institutions around the world conduct research projects that are focused on obtaining carbon-based materials for tailored properties; suitable raw materials are carefully selected for this purpose [1]. Conveniently, such raw materials are denoted as precursors. Generally, any material that is characterized by a high content of elemental carbon can be assumed to be a carbon-based material precursor. A low content of volatile components and inorganic constituents make such raw materials more valuable precursors. Recently, the presence of heteroatomic functional groups in the carbon matrix opened new application fields for such carbon-based materials. In particular, carbon–nitrogen, carbon–oxygen, carbon–sulfur, and carbon–halogen derivatives are of interest because of the very specific properties that are given to the materials by the incorporation of heteroatoms, especially on the surface. A proper selection of raw materials supported by a secondary chemical modification of precursors or carbons obtained from the precursor leads to materials with expected and valuable adsorption and catalytic properties. For example, strictly defined acid–base properties and/or a hydrophobic–hydrophilic character can be achieved through a proper manufacturing protocol. The type of the precursor and the so-called activation method (type of activating agent, thermal conditions, and the duration of the activation process) significantly affect the physicochemical properties of the most practically applied carbon-based materials, i.e., activated carbons. In addition, these properties of the already obtained activated carbons can be changed in a quite wide range through the use of appropriate chemical modifications. Such properties mainly consist of introducing various types of functional groups into the structure and/or on the surface of carbon materials, which will significantly change the chemical character.

The synthesis and use of carbons with high nitrogen content in various fields is one of the most emerging recent topics. The increase of the interest in this group of materials over the last two decades is reflected by its appearance in an increasing number of publications. In particular, research on high-nitrogen carbon materials is split into two directions: (1) the search for new organic precursors with a high nitrogen content, which can be converted into N-rich carbons; and (2) the improvement of the content of nitrogen functional groups on the surface of various carbon materials. The first research direction has recently focused on renewable natural resources. Marine/freshwater feedstock attracted researchers’ attention because of its easy accessibility, low price, continuous natural renewal, and almost unlimited volume.

### 1.1. Methods for Manufacturing Nitrogen-Doped Carbons

Activated carbons can generally be described as a solid material with a predominant content of carbon atoms, which have a high surface area and a well-developed pore structure. The developed pore structure (total pore volume and suitable pore size distribution, or PSD) enables several applications of such materials, including a selective gas or liquid phase adsorption and/or heterogeneous catalyst design. In addition to the well-developed structural parameters, the term “activated” may be among others that are attributed to the chemical heterogeneity of the surface beside some other features (surface functional groups and/or heterogeneous clusters). Chemical surface heterogeneity considerably affects the application of these activated carbons. However, the surface properties of carbons, even after intensive chemical surface modification, should be combined with appropriate structural parameters. These two features are the decisive factors in the case of possible applications. Therefore, the tailoring of structural parameters should be the main aim in the manufacture of activated carbons besides the tailoring of the chemical state of the surface.

Unique physicochemical properties, and thus a wide spectrum of possible applications of activated carbons modified with nitrogen, are the reason for undertaking the differentiated attempts towards an effective insertion of N atoms into the carbon matrix. According to some literature reports, four general strategies are well established in aiming at the enrichment of activated carbons with nitrogen [2]:

1. Carbonization of a nitrogen-rich natural raw material;
2. Carbonization of a nitrogen-rich synthetic material;
3. Secondary nitrogen enrichment of a previously produced carbon material in the gaseous phase at a higher temperature;
4. Secondary nitrogen enrichment of a previously produced carbon material in the liquid phase by impregnation with nitrogen-rich compounds, then and reheating the material.

Methods 1 and 2 lead to the enrichment of carbons with nitrogen in their whole mass, whereas methods 3 and 4 predominantly deliver nitrogen atoms to the surface. In some studies, a combination of methods 1 or 2 with 3 or 4 was described (the carbonization of poly(acrylamide-acrylate) (PAN)-based fibers was followed by a secondary oxidation with nitric acid [3]). The presented methods are not equivalent, because the type and amount of surface nitrogen groups depends on many agents, mainly the type of the raw material, the type of nitrogenation agent, and the temperature.

#### 1.1.1. Critical Evaluation of Synthetic Precursors as Candidates for Thermal Conversion into Nitrogen-Rich Carbons

The carbonization of precursors with a low nitrogen content leads to porous carbons containing trace amounts of nitrogen. Therefore, the manufacture of intensively doped nitrogen-doped carbons should be based on the carbonization and activation of precursors, which contain a high amount of nitrogen moieties. As mentioned, many manufacturing protocols were focused on synthetic polymers, such as: polyacrylonitrile, polyamides, polyimides, polyvinylpyridines, polyaniline, acetonitrile, and polypyrrole, as well as melamine–formaldehyde and urea–formaldehyde resins. Synthetic precursors are out of the scope of this review, and will not be presented in detail. Some general remarks are only made just to show that such a possibility can be exploited.

Until now, the thermal processing of synthetic precursors was performed in oxygen-free conditions. The synthesis of nitrogen-rich carbon materials from this type of precursor results in carbons having favorable parameters, such as the developed specific surface area (S_BET_) and a relatively high content of nitrogen (N). The exemplary numerical values of S_BET_ and CN for carbon materials obtained from synthetic polymers are given in Table 1.

Often, a plain carbonization of synthetic precursors yields non-porous carbon matrixes. Therefore, some pore-generating measures must be undertaken. Li et al. [4] used commercially available silica (Aerosil-200) as a hard template and melamine–formaldehyde resin as a precursor, which was later carbonized at 1000 °C. To remove the template, the material was treated with HF. Mesoporous carbon materials with large pore sizes (greater than 30 nm) have been obtained. The idea of the hard template method assumes that nano-sized solid particles must be inserted into a precursor prior to carbonization. The precursor gets thermally converted into a carbon matrix incorporating the particles of a template. The particle can be removed by dissolving with different liquid media, leaving empty cavities in the matrix and making it porous.

The use of a solid template is necessary not only in case of synthetic precursors, it is also inevitable in case of some natural marine-derived precursors such as chitosan, chitin, fish residue, etc.

The use of urea–formaldehyde resin also appeared in the paper of Drage et al. [5], where the authors activated the material with K_2_CO_3_ as a template. Raymundo-Piñero et al. [6] prepared nitrogen-containing carbon fibers by means of a petroleum pitch at a low softening temperature and a nitrogen-rich melamine resin filler, of which content varied up to 45 wt %. The application of melamine resin for the fabrication of N-rich carbons was also undertaken by Firest et al. [7]. Duan et al. proposed the use of polyurethane foam as a precursor [8]. The authors used polyurethane foam containing 14.39 wt % nitrogen and glutaraldehyde, which were subjected to a hydrothermal process (heated at 180 °C for 6 h). The material was then carbonized at three selected temperatures of 400 °C, 600 °C, and 800 °C, which yielded carbons of poorly or moderately developed structural parameters (S_BET_ in the range from 13 m^2^/g to 112 m^2^/g). Unfortunately, the nitrogen content decreased from 6.31 wt % to 0.71 wt % in line with an increasing surface area. This study, as well as all of the others mentioned in this chapter, indicates some phenomena that are also typical from the carbonization of marine/fresh water precursors. These are: carbonization temperature range (typically 600–900 °C, precursor-to-product nitrogen transfer efficiency (typically below 40%), the high probability of insufficiently developed structural properties (surface area below 10–20 m^2^/g), and the decrease of nitrogen content upon the increase of the carbonization temperature.

A similar attempt was targeted at the carbonization of the melamine foam used for the production of washing sponges [9]. Machnikowski et al. [10] demonstrated a series of activated carbons with different nitrogen contents synthesized by the pyrolysis of nitrogen-containing polymers (polyvinylpyridine and polyacetonitrile) and their mixture with tar. Pollak et al. [12] investigated the carbonization of polyimide (commercial name Kapton) mixed with ammonium chloride (at 1000 °C). Li et al. [13] investigated H_2_SO_4_-doped polyaniline subjected to carbonization and activation processes. The obtained carbon had a content of nitrogen of up to 10.89 at %., and had a well-developed surface area of up to 514 m^2^/g. It was found that the N-5 and N-6 groups were dominant over the quaternary-N (N-Q) and pyridinic N-oxide (N-X) ones. Other authors [14] investigated block polyaniline premixed with a hard template. The concept was modified by using polyaniline nanotubes instead of a block polymer [15]. Rozlivkova et al. [16] also received nitrogen-containing carbons by the carbonization of polyaniline. The process was carried out at 650 °C. Stejksal et al. used polyaniline nanoparticles [17]. The polyaniline particles were supplemented with polyvinylpyrrolidone or silica nanoparticles as soft and hard templates, correspondingly. Garsuch et al. [18], using zeolite and silica gel as a template, and polypyrrole as a carbon phase precursor, received mesoporous activated carbons containing nitrogen in the range of 5.8–9.9 at % Cong et al. [19] used polypyrrole and silica. After removing the SiO_2_, a three-dimensional structure consisting of thin carbon walls remained. Lezanska et al. [20] received a CMK-8 carbon replica using pyrrole and FeCl_3_ as a catalyst for the polymerization reaction. The obtained carbons contained a relatively high amount of nitrogen of 4.31–7.26 wt %, mainly in the form of pyridine and quaternary nitrogen. Another option was the use of polypyrrole as described in the papers of Fuertes et al. [21] and Yang et al. [22], in which silica was used as a template, and received mesoporous materials with a nitrogen content of 5.5 at % and a surface area of 1560 m^2^/g. Xia et al. [23] described the possibility of an acetonitrile conversion to the carbon matrix by means of various silicas (SBA-12, SBA-15, MCM-48, MCM-41, and HMS), which are used like hard templates. László et al. [24] manufactured nitrogen-rich carbons from polyacrylonitrile, poly(terephthalate), and cellulose, which were activated by heating (up to 900 °C) in the flow of mixed steam and nitrogen. Lu et al. [25] and Kruk et al. [26] also investigated polyacrylonitrile as a precursor of the carbon phase with the addition of silica as a hard template in order to obtain ordered mesoporous carbons. An unconventional synthesis of nitrogen-rich carbons employed hydrated disodium ethylenediaminetetraacetic acid (EDTA), which, for example, was annealed under nitrogen at a temperature in the range of 500–900 °C for 2 h [27]. In recent years, the first papers on the use of metal–organic framework structures (MOFs) as precursors of nitrogen-rich carbon materials have appeared. In 2014, Xiao et al. [28] described the synthesis of microporous carbons from MOF structures containing carbon of 68 at % and nitrogen of 25 at % after carbonization.

The above review on synthetic precursors concludes that beneficial values of S_BET_ and CN_%_ are achievable for the same raw material, provided a suitable manufacturing scenario is applied. Despite the methods and the precursor under review, all of the reported studies do not take into account the expected high production costs. Similar doubts may arise in the case of the so-called enrichment methods, which should increase the nitrogen content. Although their undisputed advantage is clear, economic and environmental issues remain unclear. Typically, nitrogen species contained in the precursor pass to the carbon matrix, but a part of it is released during heating in the form of volatile and liquid low molecular products (which are potentially hazardous). The high cost of synthetic polymers and/or the low conversion efficiency of the polymer into carbon are the main barriers to popularizing such approaches to the synthesis of high-nitrogen carbons on a scale that is larger than the laboratory. The above shortcomings of synthetic precursors can be in part omitted when renewal precursors are of natural origin. This type of precursors may be disputed in two general categories: natural but non-marine or non-freshwater origin (mainly plants and plant fragments), and real marine/freshwater feedstock.

#### 1.1.2. Critical Evaluation of Non-Marine Natural Precursors as Candidates for Thermal Conversion into Nitrogen-Rich Carbons

Precursors of non-marine origin will be briefly presented in terms that demonstrate their differences and similarities to marine feedstock. Generally, some of these precursors may suffer from a low nitrogen content. Keeping in mind the limited efficiency of the precursor-to-product nitrogen transition, such precursors are expected to yield carbon materials of very limited nitrogen content. Such precursors have carbon content that is high enough to produce the carbon matrix. Thus, some supporting measures must be undertaken to insert additional nitrogen atoms into the carbon matrix.

The use of this type of raw material is rational by economic aspects, since a reduction in production costs is expected due to the management of inexpensive and widely accessible biomass. Another important element is the pro-ecological aspect, since these methods rely on the management of a significant amount of organic waste.

##### Examples of Plant-Derived Precursors

Braghiroli et al. [29] investigated natural tannins dissolved in an ammonia water solution, which were subjected to different processing manners. Tannins dissolved in the ammonia solution were directly subjected to the hydrothermal process, yielding a higher nitrogen content of 7.9–9.4 at % in the resulting materials. Obviously, ammonia served as a nitrogen enrichment agent. Zhang et al. carried out the ammonization of sucrose (which has a very low content of nitrogen) using hydrothermal carbonization in the presence of ammonia at temperatures of 160 °C, 180 °C, and 200 °C, which was followed by carbonization at 900 °C [30]. Ammonization was necessary in order to enrich the carbon matrixes in nitrogen. The use of soy-derived wastes was described by Thote et al. [31], and supplemented by activation with ZnCl_2_ and CO_2_. The received carbons had a surface area that was equal to 811 m^2^/g, which was comparable to commercially available activated carbons having only traces of nitrogen (nitrogen content was estimated by the Kjeldahl method—0.65 wt %).

Plant-derived low-quality brown coal and derived humic acids were studied, too [32]. The carbonization and activation of these precursors allowed obtaining mesoporous nitrogen-containing carbons (2.4 wt %) with a specific surface area of 557 m^2^/g. However, the natural nitrogen content in brown coals is as high as 2–3%. Li et al. [33] worked out waste corn cobs after primary agricultural processing. One-step synthesis yielded nitrogen-doped carbon materials. X-ray photoelectron spectroscopy (XPS) analysis mainly confirmed the presence of pyridine groups. The total nitrogen content did not exceed 3.98 wt %. Natural nitrogen content in corn cobs may even reach 5–7%. Thus, this plant-derived precursor can be described as nitrogen-rich.

A controversial solution was proposed by Kim et al. [34], who carbonized silk fibers (protein-rich materials) with some activating agents. The method can be doubtful, since the cost of silk is generally high, and is silk is in strong demand by other industries.

Lua and Guo [35] studied the carbonization of oil palm followed by CO_2_ activation. Depending on the temperature and the activation time, they received nitrogen-rich carbons with a nitrogen content of 1.0–4.2 wt %. Xu et al. in 2014 [36] investigated other food product derivatives, i.e., rice husks. Another possibility of obtaining mesoporous activated carbons (in the form of flakes) is the use of pomelo mesocarp [37]. Pomelo treated with CaCl_2_ and urea, carbonized at 800 °C for 2 h, contributes to materials containing 9.12 wt % of nitrogen and a surface area equal to 974 m^2^/g. The latter study proves again that intensive nitrogen enrichment such as 9.12 wt % is achievable, provided that additional nitrogen carries are added to the main precursor.

##### Examples of Animal-Derived Precursors

Very recently, a new trend has been observed in this field, namely the use of animal-derived raw materials. These animal-derived precursors may be nitrogen-rich, since they are often built from amino acids, proteins, peptides, etc., and may contain other nitrogen species. In regard to this, some animal derived precursors resemble marine or freshwater feedstocks. Gelatine is a typical precursor of this kind. Gelatine’s average nitrogen content is about 16%, which indicates that it can be a promising precursor of nitrogen-doped carbon. In 2012, Xu et al. [38] used this inexpensive, commercially delivered, environmentally-friendly and renewable precursor, activating it with KOH. They received typically microporous carbons with an extremely high surface area of 3012 m^2^/g. However, the nitrogen content, which was equal to 0.88 at %, was low. This shortcoming was omitted by Olejniczak et al. [39], who also manufactured nitrogen-rich mesoporous carbons from gelatin supplemented with colloidal silica as a hard template. The obtained materials were mesoporous, and had a very high total pore volume of up to 4.38 cm^3^/g. A high nitrogen content was preserved of up to 10 wt %. Qu et al. [40] supplemented gelatin with SBA-15 as a hard template. They also received ordered mesoporous carbon materials. The interest in gelatin and carbon materials derived from the proven applicability of such carbons for the manufacturing of electrodes for lithium-ion batteries, supercapacitors, or as catalysts for oxygen reduction reaction (ORR) in fuel cells. For example, Schnepp et al. [41] applied Fe_3_C, MgO, and gelatin (as a nitrogen source) for the preparation of trimodal porous materials that are capable of efficiently reducing oxygen (ORR). Nam et al. [42] used carbons obtained from gelatin with some admissions of Ketjen Black, which is aimed at an increased conductivity of the obtained materials.

##### Examples of Microorganisms as Precursors

Water is the natural environment for the growth of bacteria. These organisms have recently been considered (besides plants and animals) as the precursors for the fabrication of nitrogen-doped carbons. The reasoning for this is somewhat obvious, since bacteria contain plenty of nitrogen-containing compounds. There is always a realistic chance for the transfer of such bonded “organic” nitrogen to the carbon matrix after the thermal degradation of bacteria. Pyrolysis of the “organic” precursors (bacteria) has encouraged researchers around the world to manufacture heteroatom-doped carbons without complicated protocols involving hazardous heteroatom-containing reagents. Following this general trend, Wang et al. demonstrated that N and O-doped carbon catalysts could be fabricated from gram-positive bacteria, i.e., *Bacillus subtilis*. The synthesis required chemical activators such as ZnCl_2_ or KOH [43]. Similar carbon materials can be obtained by a direct pyrolysis of gram-negative bacteria such as *Shewellea oneidensis* [44]. The carbons had catalytic properties in the oxygen reduction reaction (ORR). *S. oneidensis* is widely distributed in natural environments, including marine, freshwater, and sediments [45]. In the manufactured carbon, a variety of heteroatoms occurred, including N, P, S and Fe, which were distributed homogeneously on the carbon matrix [46,47,48]. As for nitrogen, the element was bonded to carbon matrixes as pyridinic and graphitic nitrogen. The total nitrogen content decreased from 2.58% to 2.26% with an increasing pyrolysis temperature.

The *S. oneidensis* case is not a sole synthesis involving bacteria. Zhou et al. investigated the bacteria-assisted process of gold nanoparticle insertion into a nitrogen-rich activated carbon (atomic content of nitrogen ca. 3.6%). This goal was achieved by means of *Pycnoporus sanguineus* [49]. Gold nanoparticles were embedded in a nitrogen-doped carbon matrix due to its bioreduction by microorganisms.

In summary, the use of natural-derived raw materials is justified both economically and ecologically. Some of the precursors are just wastes, which always must be somehow treated. Conversion to nitrogen-doped activated carbons may be one of these methods. Such obtained activated carbons may have a well-developed porous structure, very high surface area, and a high nitrogen content, usually of about 10 wt % in the case animal-derived precursors built of nitrogen-containing species such as amino acids, proteins, peptides, etc. In the case of less nitrogen enriched natural precursors, other nitrogen-rich additives (ammonia, urea, etc.) must be applied. The presented successful transformation of gelatin should encourage the search of animal-derived precursors. Marine and fresh water feedstock is a promising candidate for this purpose.

## 2. Nitrogen-Doped Carbon Materials Obtained from Marine-Derived Feedstock

Marine-derived feedstock presents important benefits, such as abundance, morphological and structural variety, and the presence of multiple elements. Much of the potential marine-originated feedstock contains such desirable components as amino acids, proteins, peptides, etc. Such characteristics make them successful candidates for the preparation of heteroatom-doped carbons, and particularly nitrogen-doped carbons. Most of the nitrogen species bonded to carbon matrixes are derived from the –NH_2_ group in amino acids/proteins. Water (sea and/or fresh water) is a natural environment for the growth of microorganisms. Phytoplankton are the self-feeding components of the plankton community and a key part of oceans, seas, and freshwater basin ecosystems. Researchers are still searching for low-cost and environmentally friendly methods of transferring phytoplankton into some useful materials. Since oceans contain more half of the global biodiversity, they can be considered as a potential source of bioprecursors for nitrogen-doped carbons. Another member of oceans, seas, and freshwater basin ecosystems are seaweeds, i.e., a renewable and abundant source of carbon and other heteroatoms.

### 2.1. Nitrogen-Doped Carbon from Algae and Phytoplankton

The annual production of seaweed has been calculated to be around 2.6 million tons of red algae and 16 million tons of brown algae [50]. Before using algae as precursors, the authors mostly described the methods of seaweed purification, an example which is presented in a paper by Escobar et al. [51]. In this work, *Sargassum* spp. was washed with distilled water and ethanol to remove clay sands, dust, sediments, shells, and peddles. Then, it was filtered and pulverized until an average particle size of around 75 µm was reached. The obtained raw materials can be carbonized without any other substrates, or modified with some reagents before the carbonization process. Another researcher investigated marine microalgae as a candidate for in their encapsulated protein conversion into activated carbons [52]. The study proved that different marine microalgae contain enough nitrogen to consider this biomass as a good precursor for making other nitrogen-rich materials. Kovalenko et al. [53] and Quignard et al. [54] used algae to isolate alginate, which is a natural polysaccharide extract. Alginate was converted into a stable battery electrode material (anode) possessing a reversible capacity that was eight times higher than the graphitic ones. After these pioneering works, in the last few years, the carbonization of phytoplankton to activated carbon was successfully executed and described in some papers. Generally, the manufacture of carbons from algae may follow few specific pathways: the carbonization of pristine algae, a secondary carbonization of algae-derived char in the presence of a chemical activator, and a one-step carbonization of algae supplemented with a chemical activator. These three methods may be altered by the addition of ions and/or the admission of low molecular species having nitrogen in the structure such as melamine, pyrrole, or polypyrrole aimed at the insertion of additional amounts of nitrogen.

Nori is a popular foodstuff made of ocean algae, which is rich in proteins and carbohydrates and contains small amounts of iron and other elements besides carbon, oxygen, and nitrogen. The concept of converting nori to activated carbon was verified by Liu et al. [55], who added a supplementary nitrogen carrier, i.e., melamine, to achieve a higher content of nitrogen. Generally, after melamine doping, the nitrogen content increased from 1.42 at % to 2.63 at %. Thus, the application of a nitrogen carrier such as melamine helped to yield the desired nitrogen content in the carbon matrix. The addition of nori was beneficial for the creation of mesopores and macropores, as well as a high surface area of 538 m^2^/g.

Algae biomass (*Enteromorpha*) was proposed for the synthesis of carbons [56] with a high surface area (up to 2073 m^2^/g). Such manufactured carbon had other unique features, such as a sponge-type structure. However, a low nitrogen content was noticed (0.64–0.85 at %), while the oxygen content remained high (11.36–12.24 at %). Nitrogen-doped porous carbon nanoparticles were synthesized through a low cost, and a simple method was also presented [57]. *Chlorella pyrenoidosa* was carbonized in a temperature range from 600 °C to 1000 °C. Such carbonized algae allowed reaching moderately high surface carbons (184–482 m^2^/g), in which nitrogen was bonded to the carbon matrix differently [58].

Wu et al. [59] described the surface morphology of *Chlorella*-derived chars. The algae was the component of a more complex raw material. The fractal dimension of the chars increased upon the addition of the algae to the mass subjected to pyrolysis.

**Figure 1:**
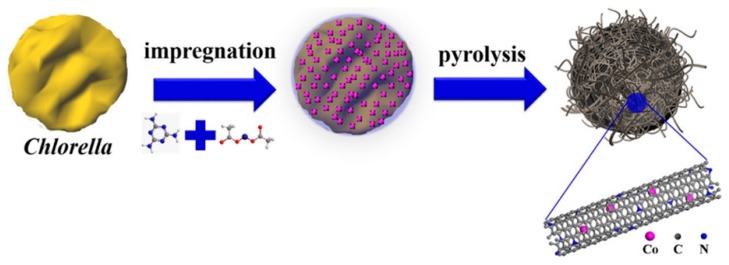
Schematic illustration of the preparation process of Co/M-*Chlorella*-900. Reprinted with permission from Wang et al. [60]. Copyright 2017, American Chemical Society.

Wang et al. [60] impregnated the mixture of *Chlorella* and melamine with a water solution of cobalt acetate, and pyrolyzed the complex precursor in an inert atmosphere (Figure 1).

**Figure 2:**
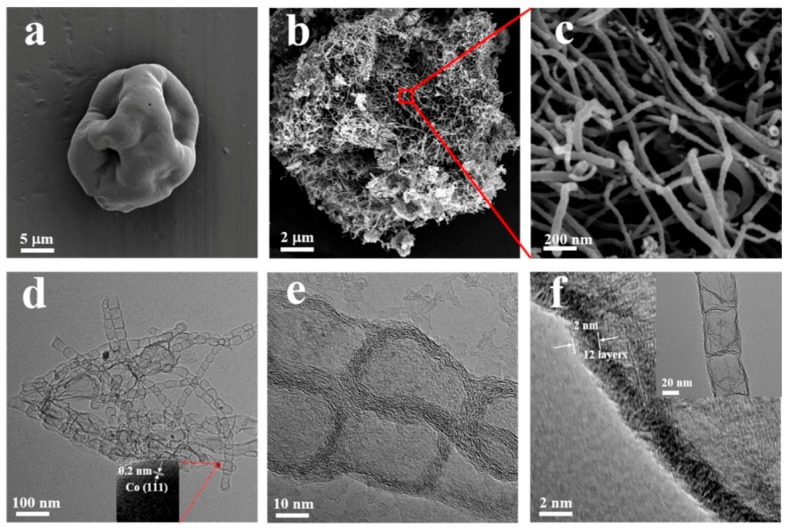
(**a**) SEM image of *Chlorella*; (**b**,**c**) SEM images of Co/M-*Chlorella*-900; (**d**) TEM image of Co/M-*Chlorella*-900; the inset is a High resolution – transition electron microscope (HR-TEM) image of a single Co nanoparticle encapsulated in N-doped carbon nanotubes (CNTs); (**e**) HR-TEM image of the joint structure of the bamboo-like CNTs; (**f**) HR-TEM image of the CNT wall; the inset shows a corresponding whole CNT. Reprinted with permission from Wang et al. [60]. Copyright 2017, American Chemical Society.

The received nitrogen-doped carbons had a hollow nest-like morphology consisting of braided bamboo-like, nitrogen-doped carbon nanotubes (up to several micrometers in length) (Figure 2). The cobalt salts played a crucial role in the transformation of *Chlorella* into the bamboo-like nanotubes and the nest-like superstructure. The specific surface area of samples obtained by carbonization at 900 °C was generally high: cobalt/melamine/*Chlorella* precursor—728 m^2^/g, melamine–*Chlorella* precursor—489 m^2^/g, cobalt–*Chlorella* precursor—604 m^2^/g, cobalt–melamine precursor—236 m^2^/g and pristine *Chlorella* precursor—157 m^2^/g. High-resolution N1s spectra confirmed the presence of four surface nitrogen-based species: N-pyridinic, N-pyrrolic, N-graphitic, and oxidized N.

Hencz et al. [61] synthesized a hierarchical porous and nitrogen-doped carbon from seaweed. The dried seaweed was supplemented with a NaCl pore gene and subsequently annealed in a tube furnace at 800 °C for 2 h under an Ar atmosphere. After template removal, valuable surface parameters were achieved: total pore volume—1.48 cm^3^/g and high surface area—1510 m^2^/g.

Godavarthi et al. [62] used waste *Sargassum fluitans* seaweed as the carbon precursor, which allowed obtaining nitrogen-doped carbon dots in a hydrothermal process. The nitrogen content was relatively high (9.85 at %) in comparison to other synthesis attempts.

Song et al. [63] reported on a template-free pyrolysis of an easily available natural seaweed, i.e., *Undaria pinnatifida*. Interestingly, the carbons obtained in this way contain heteroatoms, such as nitrogen and sulfur, in their framework, but also possess a well-developed porous structure and a high surface area. The heteroatoms are residues of these elements that were formerly present in the seaweed. Porosity was created by the removal of salts that were inherently present in the seaweed. These essential and fundamental properties make such seaweeds a prime choice as a precursor for heteroatom-containing, highly porous carbons. Similar results were achieved by Zhang et al. [64] who obtained nitrogen and sulfur co-doped carbon dots.

Subsequently, Falco et al. [65] presented how to manufacture nitrogen-doped carbons via an effective and sustainable one-step way based on the hydrothermal carbonization of microalgae with a high nitrogen content (ca. 11 wt %). The addition of monosaccharide glucose to the reaction mixture was found to be advantageous, enhancing the fixation of nitrogen in the synthesized carbons, resulting in materials possessing a nitrogen content in excess of 7 wt %, and leading to promising reaction yields. Increasing the amount of glucose leads to a higher nitrogen retention in the carbons, which suggests the co-condensation of the microalgae and glucose-derived degradation/hydrolysis products via Maillard-type cascade reactions. The increase of the processing temperature leads to a further aromatization of the carbon matrix, which includes nitrogen-containing functional motifs (i.e., pyridinic and quaternary nitrogen).

Zhao et al. [66] carbonized dried algae at 800 °C for 2 h in an Ar atmosphere. After natural cooling, a black powder was obtained containing sulfur and nitrogen, besides the domination of carbon and oxygen. The carbon matrix was predominantly mesoporous. XPS studies (N1s energy) allowed distinguishing three basic forms in which nitrogen was bonded: N-quaternary, N-pyrrolic, and N-pyridinic (total nitrogen content—1.45 wt %) Thus, the possibility of nitrogen transferring from algae to a resulting carbon matrix was confirmed again. The manufacturing scenario did not involve a template; thus, the surface area was not impressive (ca. 100 m^2^/g). Some suggestions on the unstable bonding of sulfur were presented after secondary heat-treatment experiments (sulfur can block pore entries).

The last case allowed drawing conclusions on the crucial role of templates when aiming at the well-developed surface parameters of nitrogen-rich carbons made from algae. Inicka et al. [67] investigated *Chlorella vulgaris* supplemented with nano-sized CaCO_3_ powder as an activator. The authors proved that surface parameters such as nitrogen content, surface area, and pore structure were adjustable through a proper selection of carbonization temperature and the amount of the activator used. However, a chemical interaction of the carbonate activators was not assumed. A comparison between the pore size and the grain size of the CaCO_3_ activator powder allowed to conclude that such activators played a role of an inert pore-genic substance. After removal, the substance (etching in HCl acid) disappeared and left empty cavities in the matrix, i.e., mesopores.

**Figure 3:**
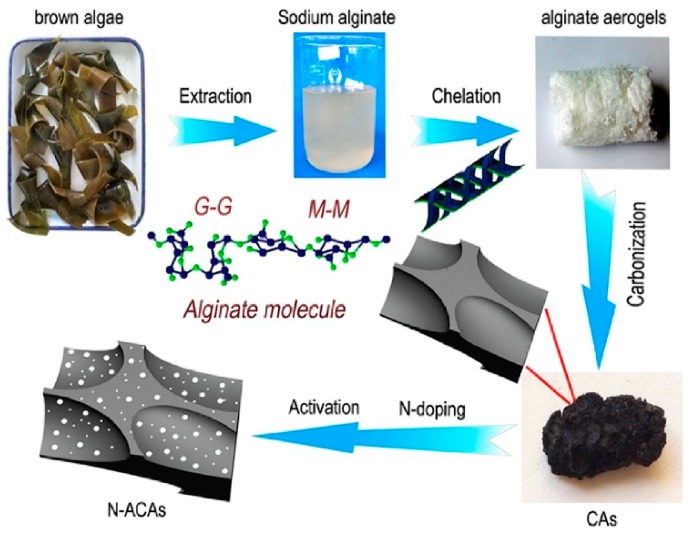
The synthesis process of N-doped carbon aerogels (N-ACAs) by pyrolysis of alginate aerogels. Reprinted with permission from Zhang et al. [68]. Copyright 2017, Elsevier.

The activation of algae-derived carbons can also be performed by means of activators, which chemically interact with the precursors and/or an already created non-porous carbon matrix. Zhang et al. [68] proposed a multi-stage procedure to obtain N-doped carbon aerogels. In the first step, the biomass, i.e., brown algae, was turned into alginate aerogels through a freeze-drying process. Then, the aerogels were carbonized at 500 °C for 1 h and heated to 800 °C for 2 h under an N_2_ atmosphere. In the last step, the obtained chars were mixed with KOH and heated again to 750 °C in the nitrogen stream (Figure 3).

**Figure 4:**
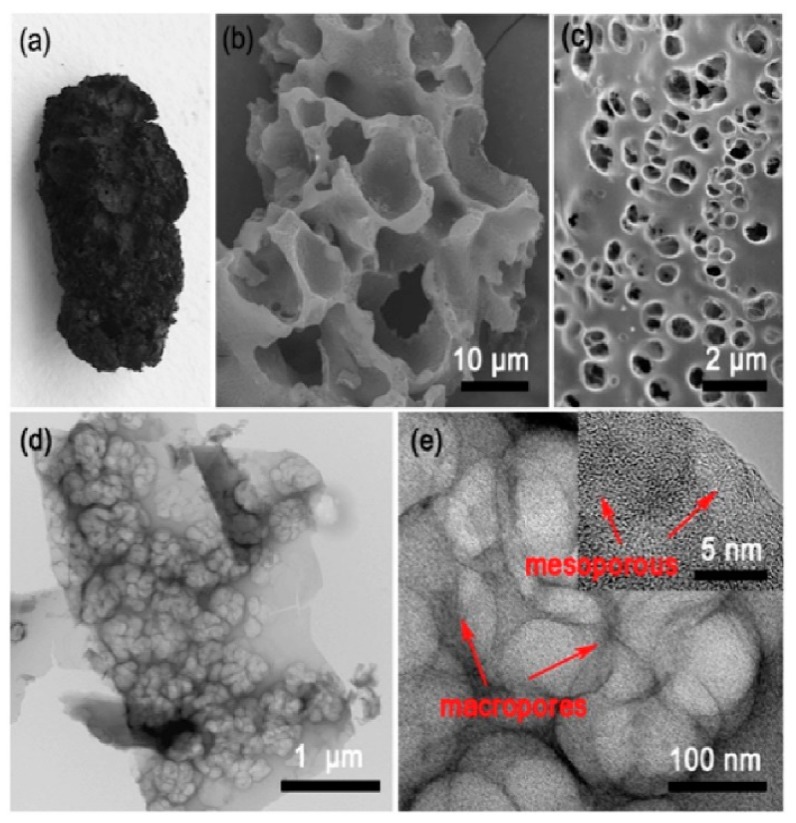
(**a**) The optical images of N-ACAs; (**b**) SEM images of N-ACAs and its amplified SEM image (**c**); (**d**,**e**) TEM and HRTEM image of N-ACAs. Reprinted with permission from Zhang et al. [68]. Copyright 2017, Elsevier.

The favorable algae-dived char/KOH mass ratio was 3:1. Additionally, to improve the content of nitrogen, the obtained carbons were treated with ammonia at 600 °C for 1 h. All of these measures allowed obtaining a high Brunauer-Emmett-Teller (BET) surface area of 2136 m^2^/g. Extended XPS studies revealed the chemical environment of nitrogen atoms, which predominantly occurs as pyridinic, pyrrolic, and quaternary nitrogen. Figure 4 shows the SEM and TEM images of the obtained N-doped carbon aerogels (N-ACAs). Apparently, the N-ACAs three-dimensional (3D) structure of interconnected pores.

**Figure 5:**
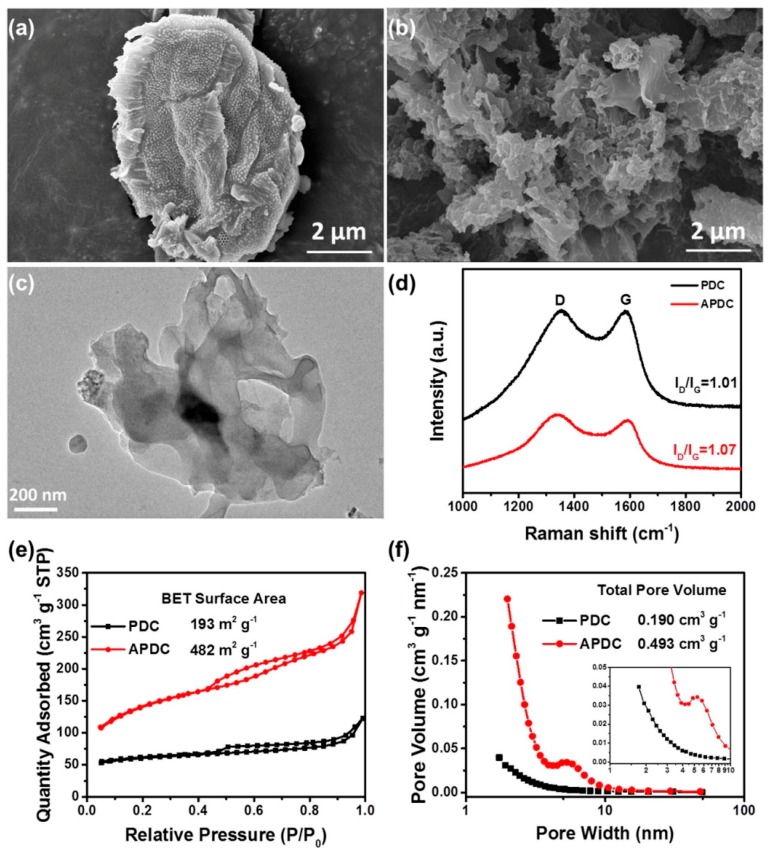
SEM images of (**a**) phytoplankton and (**b**) APDC; (**c**) TEM image of APDC; (**d**) Raman spectra of PDC and APDC; (**e**) N sorption isotherms of PDC and APDC at 77.4 K; (**f**) of pore size in PDC and APDC calculated from N sorption isotherms at 77.4 K. Reprinted with permission from Shen et al. [69]. Copyright 2017, Elsevier.

Shen et al. 2016 [69] also applied KOH as a chemical activator. Phytoplankton-derived chars were mixed with KOH (activator-to-char ration of 1:4) and heat treated. The key difference consisted in the lack of secondary nitrogenation by means of ammonia. The specific surface area was lower than the one achieved by Zhang (482 m^2^/g). As shown in Figure 5a, the scanning electron microscopy image of the phytoplankton reveals that it has a fold-shaped and uneven surface. Moreover, the transmission electron microscopy (Figure 5c) reveals a typical three-dimensional fractal nanostructure. The Raman spectra (Figure 5d) of the materials clearly showed the existence of the well known graphite sheets (G-bands) and disorder induced graphite layers (D-bands) of activated carbon materials. Figure 5e shows N_2_ adsorption isotherms being sorption isotherm of the type IV (IUPAC). The pore size distribution (Figure 5f) determined by Barrett–Joyner–Halenda (BJH) method indicated the existence of abundant micropores and mesopores.

Lee et al. [70,71] once more applied the general concept of using KOH as an activator premixed with phytoplankton (the mass ratio 5:1). The addition of the activator at an early stage of manufacturing was very beneficial regarding the final surface area ranging from 1210 m^2^/g to 2433 m^2^/g, and an extremely high nitrogen content of up to 21.8 wt %. A similar manufacturing protocol was described [72] for cyanobacteria application as a renewable source of carbon. The activation includes a pre-carbonization at 400 °C, followed by a secondary heat treatment at 800 °C in the presence of KOH. A high graphitization degree was reported, along with a large specific surface area of 2184 m^2^/g. Such a high surface area was achieved for a dominating contribution of mesopores (average pore size—27 nm) to the total pore volume.

However, as reported by Escobar et al. [51], KOH activation of the dry powder of *Sargassum* spp. (mass ratio 2:1) did not automatically provide the beneficial surface and structural properties of carbons obtained in this way. Heat treatment at 600 °C for 1.5 h in an N_2_ atmosphere only allowed obtaining materials with inferior parameters (surface area after activation—only 33 m^2^/g; nitrogen content—up to 1 wt %).

KOH is not the only chemical activator applicable to microorganism-derived carbon manufacturing. Gao et al. [73] prepared activated carbon using cyanobacteria by means of two chemical activators—KOH and ZnCl_2_. The resulting carbons activated by KOH exhibited larger amounts of micropores and mesopores, and a larger specific surface area than those activated by ZnCl_2_. The S_BET_ for samples obtained with KOH and ZnCl_2_ was in the range of 970–1951 m^2^/g and 1176–1310 m^2^/g, respectively.

A less conventional concept involved an addition of metal ions to marine-based biomass, aiming at a conversion to nitrogen-doped porous carbon matrixes (Ca^2+^, Fe^3+^, Co^2+^, and Ni^2+^). The resulting material had a unique structure denoted as an “egg-box” model [74]. Thus, the carbonization of a metal–alginate mixture may lead to the synthesis of novel 3D carbon nanomaterials with multimodal pores. A similar attempt of Liu et al. [75] yielded three-dimensional Fe_2_O_3_ nanoparticles/graphene aerogels when waste seaweed biomass was the precursor along with iron salts. The BET surface area of the materials was in the range from 71 m^2^/g to 436 m^2^/g.

Liu et al. [76] presented how to make a binder-free nitrogen-doped “carbon paper” electrode for Li-O_2_ batteries. The “carbon paper” was derived from a polypyrrole/cellulose-chopped mixture by carbonating it at 1000 °C in N_2_. *Cladophora* green algae (collected from the Baltic Sea) served as a source of nanocellulose and nitrogen, which were extracted from the algae after grinding. The received carbons had a mesoporous structure (pore sizes are in the range of 1–65 nm) and a surface area of 107 m^2^/g. The chemical composition of the surface was studied by XPS. The subsequent analysis allowed determining intensive nitrogen doping of up to 7 wt %. Pyridinic and pyrrolic structures were detected as dominating nitrogen surface species N. Therefore, the surface moieties were expected to promote some electrochemical reactions.

Other algae (red algae *Polysiphonia fucoides*) was reported by Nowak et al. [77] as a suitable precursor for the manufacture of electrodes for energy storage devices. The algae were carbonized after saturation with a glucose solution (900 °C for 2 h). However, the resulting nitrogen content was low—90.65 at %

### 2.2. Nitrogen-Doped Carbon from the Marine-Derived Precursor: Chitin

From a chemical point of view, chitin is β-(1,4)-2-acetylamino-2-deoxy-d-glucopyranose, and one of the most abundant organic materials in nature, the second largest amount after natural cellulose [78]. It is a natural polysaccharide that was first identified in 1811 by Branconnot as an alkali-resistant fraction that is a fungus ingredient [79]. Currently, this biopolymer is isolated from various living sea organisms. It is the main component of the outer skeleton of animals, such as crustaceans, mollusks, and insects. The secondary sources of chitin are various varieties of fungi, in which the cell wall of chitin is one of the main fibrous building materials. However, the dominant sources of chitin production are the shells of sea animals such as crustaceans, mainly crabs, shrimps, and krill [80,81,82,83]. An example is Antarctic krill (*Euphausia superba*), from which chitin is obtained as a byproduct. It occurs in three polymorphic forms: α-chitin (the most widespread, with the highest crystallinity, comes e.g., from the shells of crabs and shrimps), β-chitin (e.g., from squids), and γ-chitin (e.g., from the epithelium of stomachs of cephalopods, cocoons of the moth, cocoon threads of larvae of the spider beetle *Ptinus tectus*, larval and adult peritrophic membrane of adult locust, cockroach, mantis and dragonfly silkworm larva, and in sawfly larvae). These differences are related to the arrangement of the chains in the polymer and the presence of water molecules. Proteins and carotenoid pigments are covalently linked to the polymer chains. It is believed that the form of α-chitin is the most common and permanent form that is resistant to chemical agents. Chitin is an organized network of fibers with a crystalline structure that gives the discussed polymer the appropriate stiffness and strength [84,85].

As mentioned, plenty of marine/freshwater organisms beside terrestrial ones may be a source of chitin. In general, chitin occurs in nature in three crystalline forms i.e., alpha, beta, and gamma chitin, which differ due to their crystalline structures and some physical/chemical properties [86]. Chitin was discovered to be a constituent of marine sponges by Ehrlich et al. [87]. Szatkowski and Jesionowski reviewed differences between spongin and chitin-based sponge skeletons [88]. Younes and Rinaudo reviewed the preparation of chitin and chitosan from marine resources in relation to the structure/properties of such received biomaterials [89].

Kaya et al. [86] investigated properties of chitin obtained from different precursors: γ-chitin (from the cocoon of the moth), α-chitin (from exoskeleton of a freshwater crab), and β-chitin (from cuttlebone of a common cuttlefish) were compared. The analyzed forms of chitin (α, β, γ) provided a more detailed insight into the physicochemical nature of these three allomorphic forms. Wysokowski et al. [90] proved a remarkable similarity in structure between the α-chitin obtained from invertebrates, and chitin found in the fibers of the marine sponge. Alpha and beta chitin are still under an intensive investigation, while gamma chitin is less studied despite some very original structural features (every third layer has an opposing direction relative to the two preceding ones) and flexibility.

The structure of chitin as a precursor of carbon matrix may generally influence the structural properties of the resulting carbon material. This aspect of chitin application for the fabrication of N-rich activated carbons has not been clearly pointed out in most of the publications dealing with the synthesis of such materials. However, some research on chitin application (of different types and origins) for the synthesis of chitin composites may deliver some solution of the problem. Wysokowski et al. [91] synthetized beta-chitin/ZnO nanostructured composites (chitin scaffolds were isolated from the skeleton of marine cephalopod *Sepia officinalis*). This composite was not turned into a carbon matrix by carbonization, but such a manufacturing step is worth investigating. Such a conversion possibility was demonstrated by Szatkowski et al. [92], who carbonized a proteinaceous spongin template for the fabrication of 3D structured MnO_2_-based composite. Fragments of carbonized spongin scaffolds were soaked in a KMnO_3_ solution, and then transferred to a hydrothermal reactor. The carbonized spongin, whose structure resembled the original spongin, was covered by a layer of MnO_2_ nanocrystals. The composites were tested as a positive electrode in an asymmetric electrochemical capacitor. The material revealed some advantages over other carbon/MnO_2_ electrodes, such as high stability after 3000 charging cycles and easy manufacturing. 

Wysokowski et al. [93,94] received alpha-chitin/hematite composites (alpha chitin was derived from *Aplysina* sponges). The novel composite had a 3D structure that was formed under extreme biomimetic conditions. Also, this material was not converted into a carbon matrix by carbonization, but after premixing with a commercial activated carbon (Norit DLC Supra), it was successfully tested as an electrode material. A similar synthesis pathway was presented by Szatkowski et al. [95]. The resulting chitin/hematite composites were tested as electrode materials (after premixing with a Norit commercial carbon) in a laboratory scale supercapacitor. Activated carbon was used as an enhancer of porosity and a specific surface area, while a more active role was assumed for the chitin-originated composite. Such synthesis approaches exploited biomimetic phenomena. It yielded specific 3D structure resembling spongin-type precursors.

Chitin and chitosan can be converted to an active carbon by carbonization as an electrode material. However, chitin sponge can be applied for the construction of electrochemical devices in a more or less pristine form. Stepniak et al. [96] used the demineralized skeleton of marine spongin *Ianthella basta* as a source of a chitinous network that later served (after some additional preparative steps) as an membrane for supercapacitors.

The pyrolysis mechanism is specific to each type of the material. However, it is possible to describe the basic steps related to the thermal destruction of the original macromolecular structure that is common to all polymeric substances. The destruction of less durable bonds in the polymer network occurs in the early stage of carbonization (about 300–350 °C) and generates free molecules of water, methanol, methane, and carbon dioxide. At the same time, carbon atoms are regrouped to stable six-membered rings. In addition, perpendicular bonds between macromolecules are formed. The creation of a cross-linked, rigid carbon structure is responsible for the appearance of the microporosity of the material. Exceeding the temperature of 500 °C increases the degree of carbonization and aromatization of the structure; the reason for these changes is the elimination of hydrogen and oxygen atoms. At carbonization temperatures above 700 °C, small packages of polycondensed carbon rings (six-membered) become visible. However, the presence of strong transverse bonds prevents their mutual ordering and the course of the graphitization process [97].

Stolarek and Ledakowicz [98] analyzed the kinetics of chitin pyrolysis using non-isothermal thermogravimetry. In the studies, they used chitin obtained from Antarctic krill. Based on the conducted studies, the authors found that an intense destruction of chitin is observed in the temperature range of 350–380 °C. This temperature is higher than for cellulose or other biopolymers described in the literature that have a similar structure. As might be expected, in the case of chitin, this is associated with a higher degree of polymerization.

Wanjun et al. [99] described the thermal destruction of chitin as a one-stage randomly initiated process, which probably is strongly controlled by depolymerization. Under nitrogen, chitin has a higher decomposition temperature than for oxidative conditions due to the high thermal stability of the acetylated side chains in the excess of oxygen.

In 2015, Qiao et al. [100] investigated the thermal destruction of chitin and chitosan (a derivative of chitin). The authors showed that chitin has a lower thermal stability than chitosan. In addition, the use of mesoporous SBA-15 and MCM-22 molecular sieves (as catalysts/templates) resulted in a 50% weight loss being observed at 229 °C and 233 °C, respectively, which are significantly lower than the corresponding temperature for the analyzed (no catalyst) chitosan sample (261 °C). The decomposition is associated to the evolution of gases like NH_3_, H_2_O, CO, and CO_2_, which can be identified by mass spectroscopy. The experimental results reveal that the obtained carbon materials lost their original chain hydrocarbon structure and were completely converted to an aromatic structure with a high carbon and nitrogen content. The relation between the course of the thermal decomposition and the deacetylation degree was examined by Qiao [101].

The carbonization of raw chitin obtained from crustaceans (containing pure chitin and some other components) and wood (containing pure cellulose, hemicellulose, lignin, and some other components) immediately leads to chars having a well-developed pore structure [102]. The reason is the occurrence of substances that act as natural soft templates in these complex materials, which are removed by gasification during the thermal processing of the raw material (oxygen-free carbonation) [103]. The release of the natural templates most probably takes place after the formation of a carbon matrix from the thermally decomposed cellulose. Hemicellulose decomposes at a temperature lower than cellulose, leaving empty cavities (pores) in the matrix.

Ilnicka and Lukaszewicz [104] described the carbonization of chitin treated with a chemical activator Na_2_CO_3_ as a method suitable for the synthesis of N-doped microporous carbons. The obtained high nitrogen-containing carbon materials have a high S_BET_ surface area, ranging from 293 m^2^/g (carbonized at 600 °C sample CA_600/A(20)) to 1346 m^2^/g (carbonized at 800 °C sample CA_800/A(60)). The total pore volume V_t_ of these materials has reached high values, ranging from 0.157 cm^3^/g (for sample CA_600/A(20)) to 0.718 cm^3^/g (for sample CA_800/A(60)). With increasing temperatures of up to 800 °C and an increase in the activator volume, S_BET_ increases to 1346 m^2^/g. For higher temperatures of 850 °C and 900 °C, the high surface area decreased to 990 m^2^/g and 915 m^2^/g for samples CA_850/A(20), CA_900/A(20), respectively. According to the IUPAC classification, all of the nitrogen adsorption isotherms for these carbons are type I isotherms with a predominant proportion of micropores and a slight share of mesopores. The primary investigation was continued in a paper of the same authors [102]. A series of activated carbons were prepared from chitin by means of phosphoric(V) acid as an activator. The results showed that all of the investigated activated carbons had a high nitrogen content in the range of 3.1–6.4 wt %. The XPS studies revealed the presence of four typical nitrogen-based surface species corresponding to: pyridinic N (N-5, 398.3 ± 0.2 eV), pyrrolic N (N-6, 400.0 ± 0.3 eV), and oxidized N (N-X, 403–405 eV). Quantitative XPS analysis proved that the N-6 nitrogen content was generally higher than that of the N-5 and N-X nitrogen atoms. Moreover, the following relationship was noticed: N-6 > N-5 > N-X.

In 2009, White et al. [105] discovered that the plain carbonization of natural inorganic (CaCO_3_)/organic (shell chitin) composites yielded a nitrogen rich-carbon material naturally templated by the mineral scaffolds. The resulting carbonaceous composite may be purified from the inorganic component by washing with acetic acid, leading to enhanced mesoporous properties.

Nguyen et al. [106] employed chitin nanorods prepared by the sequential deacetylation and hydrolysis of fibrils isolated from king crab shells. The nanorods were organized into a nematic liquid-crystalline phase. Silica/chitin composites obtained by the sol-gel condensation of silica in the presence of liquid-crystalline chitin nanorods were carbonized and etched to yield mesoporous nitrogen-doped carbon films that replicated the layered nematic organization of the nanocrystalline chitin films.

In the case of the Liu et al. study [107], three-dimensional (3D) N-doped porous carbon (NPC) materials were developed by a template-assisted (e.g., SiO_2_ spheres) high-temperature pyrolysis of shrimp-shells. Such derived N-doped carbon nanodots (N-CNs) were obtained with an overall yield of ~5%. The surface composition involved O-containing and N-containing functional groups. The elaborated method involved SiO_2_ spheres with an average diameter of ~200 nm inserted into the starting composite, which was further thermally treated in an N_2_ atmosphere at different pyrolysis temperatures. Carbonization was followed by HF acid etching to obtain 3D N-doped porous carbon (NPC) materials.

In 2016, Zhang et al. [108] reported the synthesis of Fe/Fe_2_O_3_ nanoparticles anchored on Fe-N-doped carbon nanosheets using shrimp shell-derived N-doped carbon nanodots as carbon and nitrogen sources in the presence of FeCl_3_ by the pyrolysis approach.

Also in 2016, Zhang et al. [109] used shrimp shells for the production of N-doped carbon materials. This time, the authors introduced cobalt ions. Generally, Co/CoO nanoparticles were immobilized in a carbon matrix, and hybrid Co-N-doped carbons were developed. Shrimp shells served as a precursor for N-doped carbon matrixes.

In the case of studies by Roman et al. [110], prawn shells were a raw material used for the production of mesoporous adsorbents via hydrocarbonization. The influence of the carbonization temperature rise on the basic properties of N-rich activated carbons was investigated, too.

**Figure 6:**
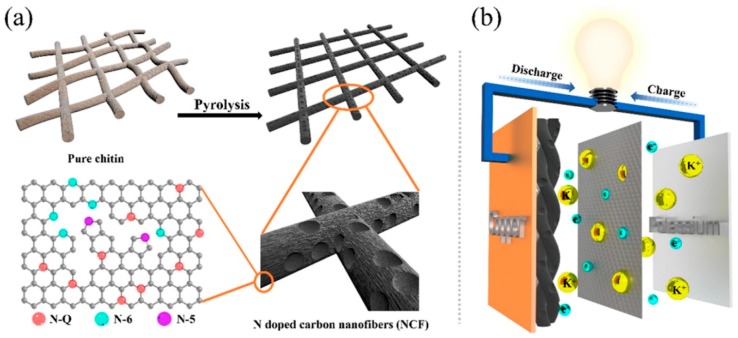
Schematic illustration for the synthesis of (**a**) the N-doped carbon nanofibers (NCFs) and (**b**) their application in potassium ion batteries (KIBs). Reprinted with permission from Hao et al. [111]. Copyright 2018, Elsevier.

Recently, Hao et al. [111] fabricated N-doped carbon nanofibers by the direct pyrolysis of biowaste chitin under Ar atmosphere (Figure 6a). Key properties for electrochemical applications (Figure 6b) such as specific surface areas, porosity and N-doped surface functional groups can be tuned by an appropriate thermal treatment temperature: 500 °C, 700 °C, 800 °C, and 900 °C.

**Figure 7:**
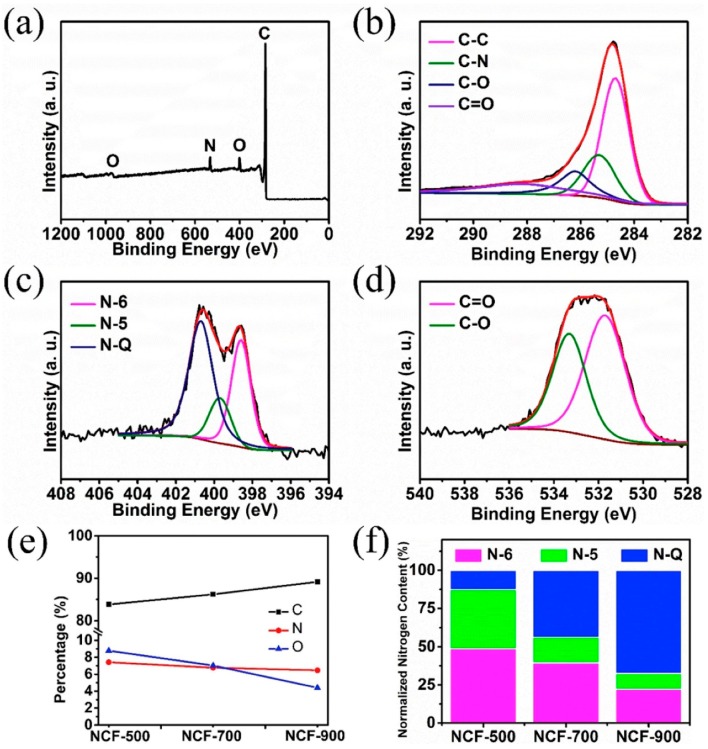
XPS spectra for the NCF-700: (**a**) The survey; (**b**) The high resolution of C 1s peak; (**c**) The N 1s peak; (**d**) The O 1s peak; (**e**) Elements (C, O, N) and their contents in as-synthesized NCFs; (**f**) Different content of N species in as-synthesized NCFs. Reprinted with permission from Hao et al. [111]. Copyright 2018, Elsevier.

As shown in Figure 7, XPS spectra contain three main elements: C, O, and N. The content of nitrogen species changed upon the increasing annealing temperature (Figure 7f) i.e., the content of N-5 and N-6 obviously decreased while that of N-Q gradually increased. N-doping of carbon matrixes generally generated more defects/current carriers and enhanced electronic conductivity. However, excessively high N-Q content may result in bad cycling performance.

### 2.3. Nitrogen-Doped Carbon from the Marine-Derived Precursor: Chitosan

Chitosan does not occur in nature in a pristine form. However, it is a derivative of chitin that is subjected to the deacetylation process. On the contrary to chitin, chitosan was not used for the manufacture of N-rich carbons until the first work of Kucinska and Lukaszewicz in 2012 [112]. This work was followed by numerous subsequent studies, which were based on manufacturing scenarios that were more or less modified the primary synthesis.

The thermal degradation of chitin and chitosan occurs randomly, causing weak bonds to crack. However, Wanjun et al. [99] showed that the thermal decomposition of chitosan under nitrogen differed from the thermal decay of chitin. Chitin has a stable initial decomposition temperature, due to the high thermal stability of the acetylated side chains that can inhibit the degradation of the chitin chains. Moreover, the activation energy of thermal degradation of chitin is usually constant. In the case of chitosan, the activation energy of thermal decomposition depends on the degree of conversion/deacetylation. Despite the deacetylation process, some residual acetylated parts are randomly distributed over the main chitosan chains. The presence of the acetylated sites increase the thermal stability of the material.

Qiao et al. [100] revealed the relation between the thermal stability of chitosan and its molecular mass, i.e., the degree of polymerization. Different chitosan samples were investigated: chitosan-5K, chitosan-50K, and chitosan-100K. A slight weight loss was observed up to 100 °C, which was commonly attributed to the evaporation of physically adsorbed water from the samples. Efficient thermal decomposition for chitosan-5K began at 140 °C, and reached a maximum at 197 °C. The completion of thermal decomposition was recorded at 330 °C. For the remaining two samples, the degradation began and proceeded at elevated temperatures. Thus, differences in the thermal behavior of chitosan could be attributed to its molecular weight or the degree of polymerization, too. Samples with higher molecular weights are more thermally stable. The relation between the thermal distribution and the degree of deacetylation has also been described by Kaczmarek and Zawadzki [101,113].

Bengisu and Yilmaz [114] have described how the oxidation and pyrolysis of chitosan can lead to the derivation of carbon fibers. The mechanism of thermal degradation of carbon during the oxidative pyrolysis of chitosan was studied by Fourier transform infrared (FTIR). It was suggested that degradation is associated with the formation of a pyranose ring accompanied by a partial dehydration and deamination. Zeng et al. [115] determined the composition of volatile compounds formed during chitosan pyrolysis. The following volatile species were identified: pyridine, pyrazine, pyrroles, and furans. Pyrazine dominated over other volatile substances. Basing on the study, the formation of furan rings was expected as a result of the dehydration of some intermediates such as the polyhydroxyacids of pyrazine. α-amino carbonyl structures that were produced as a result of chitosan degradation were generally considered to be precursors of pyrazine compounds.

In the following text, several methods for the preparation of chitosan-originated nitrogen-rich activated carbons are reviewed. The review considers the historical aspect of the synthesis approaches, including the first scientific paper and patent applications in this field. The relation between the manufacturing of nitrogen-containing carbon from chitosan and the resulting textural properties is discussed (i.e., surface area, total pore volume, and pore size distribution). The influence of the obtaining method on the nitrogen content in the carbons is additionally presented. The identified potential applications of chitosan-originated nitrogen-rich carbons are described in relation to their basic physical and chemical properties.

As mentioned above, chitosan is a nitrogen-rich polymer of a biological origin that is not directly available in nature. It is obtained by the deacetylation of a biopolymer that is present in nature, i.e., chitin. In practice, large amounts of chitin are obtained from sea flora such as shellfish, crabs, and krill (shells). However, such obtained chitin is, in fact, a composite material containing this biopolymer and other components being a natural soft template. On the contrary to chitosan, chitin is insoluble in water [116]. This is an important limitation for the transformation of chitin into nitrogen-containing carbons. The water solubility of chitosan opens new possibilities, such as the polymer premixing in the liquid phase with plenty of solid and liquid activators and modifiers. Both chitosan and cellulose show far-reaching structural similarities. Compared to cellulose, in chitosan, hydroxyl groups are replaced by amino groups; therefore, chitosan is a potential precursor of high-nitrogen content carbon materials, while cellulose is not. The second difference is the insolubility of cellulose in water and water solutions, as in the case of chitin. Some studies showed that a direct carbonation of pure chitosan and pure cellulose yielded carbonaceous materials with a very poorly developed porosity [103,112]. Therefore, some activation procedures must be undertaken in order to develop the pore structure and surface area of the carbons that are derived from these precursors.

According to the synthesis protocols announced by Ilnicka and Lukaszewicz, the application of chitosan as a new precursor for high-nitrogen carbon materials allowed obtaining carbon materials of specific properties from this precursor for the first time. The papers of these authors, which were published after 2012, developed primary ideas that were first disclosed in a patent application (2011). In 2015, the Polish Patent Office granted a patent that confirmed the novelty of the concept [117]. The patent described the preparation of nitrogen-rich carbons from chitosan by means of a carbonate activator. In subsequent stages of the research on chitosan, two new patent applications were submitted to the Polish Patent Office in 2015 [118,119].

As mentioned, some preliminary studies of Ilnicka and Lukaszewicz showed that the simple carbonization of pure chitosan leads to carbon materials with a poorly developed porosity. However, the nitrogen content was high, which allowed chitosan to still be considered a promising precursor. Therefore, a number of modifications aimed at the improvement of the structural properties of nitrogen-rich carbon materials obtained in this way were proposed and carried out. Chitosan, as a synthetic derived from chitin—contrary to the later one—is devoid of substances that could act as a natural template. Therefore, such pore-genic substances are templates that should be introduced at a certain stage of manufacturing. The key stages of the first method for the preparation of carbons from chitosan are presented in Table 2. In general, this method, called method A, can be modified from step 3 (described in Table 2) according to at least five alternative scenarios (Table 3).

Table 3 presents a brief description of these five modifications, which are denoted as scenarios B, C, D, E, and F. Method E confirmed the possible biological activity of carbons manufactured in this way. 

The conducted research allowed stating that the proposed activation measures (Na_2_CO_3_, CaCO_3,_ ZnCl_2_, H_3_PO_4_ etc.) lead to an effective improvement of the structural parameters of carbons obtained from chitosan in comparison to the material obtained after the carbonization of the non-activated material. Method A before all aimed at the development of structural parameters through the use of Na_2_CO_3_ as a removable template and chitosan as a carbon phase precursor. Shortly after mixing the basic components (chitosan and water), the chitosan-based smeary paste was pretreated with HCl, aiming at a partial depolymerization and protonation of amino groups. HCl addition allows admitting more water (water-based solutions as well) and a homogenization of the paste to a gel consistency. The structural parameters of carbons obtained in this way were influenced by the volume and concentration of the Na_2_CO_3_ solution and the temperature of the carbonization process. Beside the development of pores in the carbon matrix, method A was aimed at increasing the specific surface area. The obtained carbons were microporous in general, and their S_BET_ specific surface area was in the range from 441 m^2^/g to 1148 m^2^/g, while the nitrogen content reached values of up to 6.5 wt % [112,122]. The primary activating agent (Na_2_CO_3_) allowed reducing the temperature of the process as well as the time necessary to develop the porous structure. In general, activation with the Na_2_CO_3_ solution leads to the production of microporous carbons without a significant share of mesopores (methods A and U). Similarly, activation with ZnCl_2_ (method B) leads to the production of microporous carbons that are enriched with nitrogen in the range of 4.5–7.5 wt %. Changes in the nitrogen content significantly depended on the carbonization temperature.

Modifications B, C, and D were aimed at obtaining better structural parameters than those for the standard method A, such as a further development of the surface area, increasing the total pore volume, and increasing the share of mesopores. The purpose of modification U was to increase the nitrogen content, especially when using a carbonization temperature above 800 °C, when the nitrogen content intensively decreases due to the thermal decomposition of nitrogen functional groups [125]. The research has shown that the introduction of additional amounts of nitrogen, i.e., the C_N%_ increase, is possible by applying method U based on the use of the urea solution. Although the C_N%_ content increased even above 13 wt %, there was also a deterioration in the structural parameters, a decrease in S_BET_, and a decrease in the total pore volume V_t_ (Table 4). These parameters were reduced two to threefold in comparison to the analogous carbon materials obtained without using method U.

Modification E was based on the admission of the solution of copper ions; it allowed receiving materials with new fungicidal and bactericidal properties. These properties have been confirmed in microbiological tests on selected bacterial and fungal strains. It is not necessary to develop the surface area in order to maintain these properties. High biocidal activity has been reported for such materials (Cu^2+^ ion addition) with a developed surface area (up to 1159 m^2^/g—obtained according to method B), as well as having a small surface area (102–123 m^2^/g—obtained without the use of an activator ZnCl_2_). The presence of chitosan at early manufacturing stages causes the adsorption of Cu^2+^ ions in this precursor, which is possible thanks to the presence of amino groups in chitosan. The carbonization of chitosan modified in this way leads to the formation of nanocrystallites of copper(II) oxide, copper metal, and, above all, copper(I) oxide, which are homogeneously dispersed in the carbon matrix, which is crucial for the biocidal activity.

Table 4 summarizes the basic properties of the high-nitrogen carbon materials obtained according to the A–E methods. These methods were developed and applied for the first time in the period 2012–2016.

The beneficial properties of high-nitrogen carbon materials obtained by using chitosan as a precursor and A–U methods contributed to the widespread use of chitosan in the synthesis of carbon materials. One can even speak of a significant acceleration of research papers dealing with the synthesis of nitrogen-rich carbon chitosan in 2015 and 2016.

The use of chitosan as a raw material has a number of advantages over synthetic precursors. Among the advantages, besides physicochemical properties such as high nitrogen content in the resulting carbons, one should mention the renewable nature of this material. In the 2012–2016 period, the number of papers and other scientific announcements focused on chitosan increased significantly. The increase may be associated with the outstanding electrochemical properties of chitosan-derived carbons, which are well suited to electric power generating/storage devices such as an electrode material in supercapacitors, fuel cells, and metal–air batteries. Besides, nitrogen-rich carbons, including the chitosan-derived ones, can be applied as effective adsorbents of heavy metal pollutants from drinking water and other liquids, as well as for the accumulation of CO_2_. Most of the papers that appeared in 2015–2016 describe such emerging applications. Particular attention is paid to electrochemical applications, taking into account the ability of these carbons to catalyze the ORR. The most typical applications of chitosan-derived carbons are summarized in Table 5.

Table 5 contains information on the manufacturing protocols, showing some similarities to the previously discussed methods A–U. The analysis of the references allowed pointing out the dependence of the electrochemical activity of such carbon on the activation method, as well as the content of nitrogen groups and the method of thermal treatment. A new manufacturing scenario was proposed as a two-step procedure involving the secondary activation of a virgin char as a product of chitosan carbonization. For example, such a raw chitosan-derived char can be calcined with strong bases such as KOH and/or activated with CO_2_ in the gas phase. The use of a secondary activation leads to a significant development of the specific surface area up to the range of 3500 m^2^/g. However, very often, the nitrogen content in materials obtained in this way is not specified. For example [137], chitosan, after being converted to a water gel, was carbonized to obtain a non-porous carbon material (first stage). In the second stage, the carbon was soaked in contact with CO_2_ at an elevated temperature. This allows obtaining a carbon-type electrode material suitable for the construction of a supercapacitor. In other instances, raw chitosan powder was subsequently turned into a water gel, carbonized to a non-porous material, and finally heated in contact with NaOH or KOH. Such manufactured materials were useful for the accumulation of hydrogen or as an electrode in supercapacitors [127,133,134,136].

The basic method A was followed in some studies, usually adding some modifications. Lezanska et al. [129] applied activators ZnCl_2_, KOH, and CO_2_. In this way, the contribution of mesopores to the total pore volume increased. These carbons exhibited a high surface area of up to 1770 m^2^/g.

**Figure 8:**
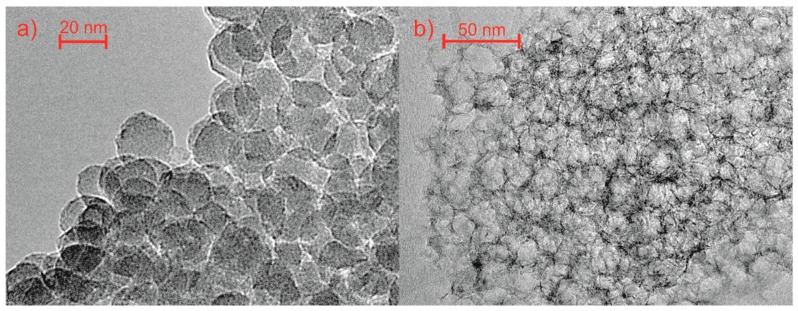
HR-TEM images of the silica–chitosan composite (**a**) and CAS40-1.75 mesoporous carbon (**b**). Reprinted with permission from Olejniczak et al. [128]. Copyright 2013, The Royal Society of Chemistry.

Olejniczak et al. [128] synthesized nitrogen-containing mesoporous carbons using colloidal silica as a hard template and chitosan as a carbon phase precursor. The final product had an extremely high total pore volume of 4.31 cm^3^/g, which was almost exclusively attributed to mesopores. The TEM image of a silica–chitosan composite is shown in Figure 8a. The HR-TEM images (Figure 8b) revealed that the majority of pores were spherical with an internal diameter reflecting the size of the silica nanoparticles.

Guo et al. [138] used a chitosan and silica template to obtain 3D hierarchical nitrogen-rich carbons resembling a nanoflower (NCNF). The as-prepared NCNF with abundant mesoporous channels displays a surface area equal 907 m^2^/g and a pore volume of 1.85 cm^3^/g. Nagar et al. [139] encapsulated Nd_2_O_3_ domains in a chitosan-derived carbon matrix by annealing the chitosan-Nd(OH)_3_ microspheres mixture.

### 2.4. Nitrogen-Doped Carbon from Sea Animals

Many complex organisms i.e., animals, live in the marine environment and potentially may serve as a precursor for nitrogen-doped carbon materials. This concept is presented in many papers devoted to such carbons from different parts of fish. A review on this particular subject is given in a separate chapter (Section 2.5). As mentioned, the elemental content of raw biomaterials such as water animals is important, since the nitrogen content should be high in order to enable an efficient transition to the resulting carbons. The content of other elements such as phosphorus is important for the prediction of the occurrence of impurities in the carbon matrix. The elemental composition of some Baltic Sea crustacean zooplankton was studied [140]. The copepod *Acartia* sp. had a stable C and N content (48.3 C, 12.4 N). Copepods accumulating fat such as *Pseudocalanus minutus elongatus* had a higher and more variable C content (50–60%), and a lower N and P content (7–12% N, 0.6–1.5% P). The cladocerans *Bosmina longispina maritima* and *Evadne nordmanni* had a lower N content (9.3–10.8%) and a higher C:N ratio (5.1–5.7) than *Acartia* sp. Generally, the nitrogen content in the pristine sea zooplankton is high enough for its effective transfer to carbon matrixes. The animal elemental content is dependent on the structure of the zooplankton and the place of occurrence [141]. The case of Baltic Sea zooplankton allowed assuming that zooplankton occurring in other seas and oceans is also a promising candidate for the transformation to nitrogen-rich carbon.

**Figure 9:**
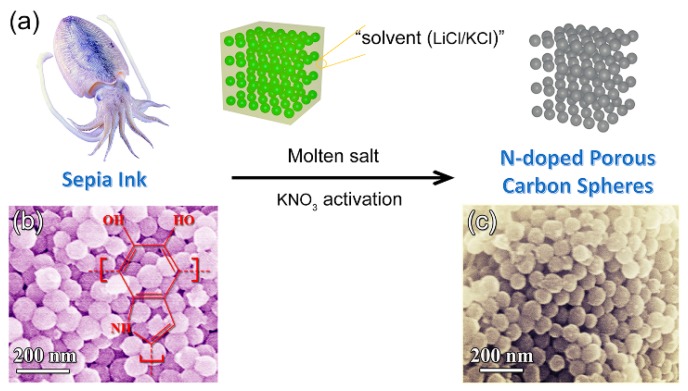
(**a**) Schematic illustration for fabricating N-doped porous carbon spheres. SEM images of (**b**) sepia ink nanoparticle and (**c**) MA-NCS. Reprinted with permission from Hao et al. [142]. Copyright 2017, Wiley.

Hao et al. [142] confirmed that biowaste sepia ink is a sustainable source for synthesizing highly porous, nitrogen-doped carbon nanospheres by a molten salt-based activation strategy (Figure 9). Sepia ink is a rich brown pigment derived from the ink sac of the common cuttlefish sepia.

**Figure 10:**
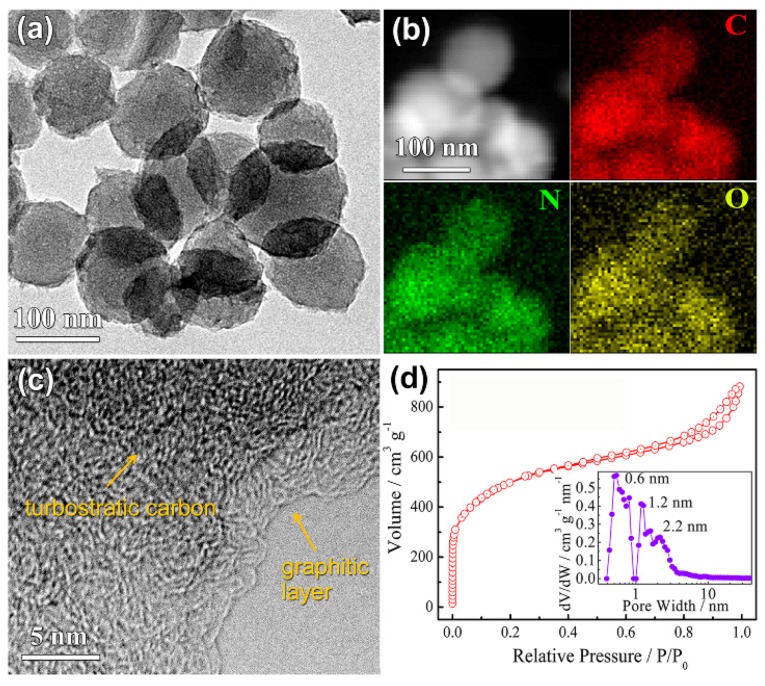
(**a**) TEM image of MA-NCS; (**b**) Scanning TEM image of MA-NCS and corresponding elemental mapping images of C, N, O; (**c**) HRTEM image of MA-NCS; (**d**) N adsorption/desorption isotherm and pore size distribution 2 (inset) of MA-NCS. Reprinted with permission from Hao et al. [142]. Copyright 2017, Wiley.

The morphology and microstructure of the as-prepared carbon materials is shown in Figure 10b. The sepia ink nanoparticle displayed a nanosphere shape and uniform size. After molten salt-based activation, carbon nanospheres (MA-NCS) (Figure 10a) still maintained a nanosphere shape, with a diameter of about 100 nm. The scanning electron microscopy with energy dispersive X-ray spectroscopy (SEM-EDX) mapping images (Figure 10b) clearly demonstrate that the N element was uniformly distributed over the entire carbon framework. High-resolution TEM in Figure 10c revealed that the carbon nanospheres consist of a well-developed amorphous structure with a partial graphitic layer. As shown in Figure 10d, MA-NCS exhibits type I isotherm characteristics, indicating the presence of a micropore-rich structure. The as-obtained carbon nanospheres had a large surface area of 1760 m^2^/g, a uniform pore architecture, and a high nitrogen content (8.6 wt %). The pore size distribution (PSD) was calculated by the non-local density functional theory (NLDFT) method from the N_2_ adsorption isotherm, displaying three main peaks at 0.6 nm, 1.2 nm, and 2.2 nm (insert in the Figure 10d).

The above discussion presented ways of synthesizing nitrogen-doped carbon materials. However, there are some doubts concerning the unlimited accessibility of these precursors and their price. Despite the scientific and technological achievements, a search for less controversial resources is necessary. Fishery and fish processing may deliver suitable amounts of inexpensive nitrogen-rich precursors.

### 2.5. Nitrogen-Doped Carbon from Parts of Fish

An extensive survey done by the Food and Agriculture Organization reveals that the worldwide capture of fish through commercial fishing in wild fisheries and fish farms amounts to 93.2 million tons and 48.1 million tons, respectively, whereas fish waste discarded in landfills or dumped at sea amounts to about 18–30 million tons [143]. Fish meat and other fish parts are rich in proteins and amino acids, making them a potential precursor for producing N-containing carbon materials. Using fish-derived precursors for nitrogen-doped carbon fabrication should be attractive in terms of price, and it definitely exploits renewable resources. Besides proteins and amino acids, fish bodies contain inorganic calcium-deficient hydroxyapatite, which is well dispersed in the organic components [144]. The presence of such inorganic components may be considered as a template embedded in an organic precursor and later in a carbon matrix. The mineral phase, i.e., a natural template, is removable, and therefore plays a pore-genic role. Furthermore, fish scales are inexpensive, and are very often treated as troublesome waste. These assumptions were confirmed by the synthesis of hierarchical lamellar carbon materials, as described in the literature [145].

Fishbone contains a significant amount of protein and fat, which can be used as a source of nitrogen and carbon in nitrogen-doped synthesis, too. Through XPS studies, Wang et al. [146] confirmed that the nitrogen content in the fishbone-derived mesoporous carbons might reach up to 6 wt %. Wu et al. [147] applied a hydrothermal treatment of fish scales and prepared nitrogen-rich photoluminescent carbon nanodots. The XPS studies proved a very high nitrogen content of 14.6%.

Yu et al. [148] investigated the acid-treatment effect on the N-doped porous carbon obtained from fish scales. They found that HCl and HNO_3_ might be employed to remove the natural template. Carbons treated with HNO_3_ had a larger specific surface than the ones after HCl etching. An opposite effect was described in an earlier paper by Kucinska et al. [112], who tried to remove carbonate templates from chitosan-derived nitrogen-rich carbons. After HCl etching, carbons treated in this way had a surface area of up to 440 m^2^/g, while HNO_3_-etched carbon had a much smaller surface area of up to 19 m^2^/g. The surface minimization was attributed to the oxidative properties of the nitric acid, which caused template removal, but also destroyed fragile (high surface) elements of the carbon matrix.

Huang et al. [149] reported a strategy of fish scale conversion to activated carbon that was capable of intensive CO_2_ capture. The synthesis involved thermal and chemical treatment in order to convert fish scales into N-rich porous carbons. As usual, the resulting nitrogen content in carbons manufactured this way decreased upon an increase in the carbonization temperature. The CO_2_ capture capacity was attributable to the pore volume (micropore volume of up to 0.76 cm^3^/g and the total pore volume of up to 2.29 cm^3^/g), and an extremely developed specific surface area (up to 3206 m^2^/g). It was assumed that quaternary nitrogen (2.90 at %) played a crucial role in the CO_2_ capture through the chemical adsorption of CO_2_ molecules.

**Figure 11:**
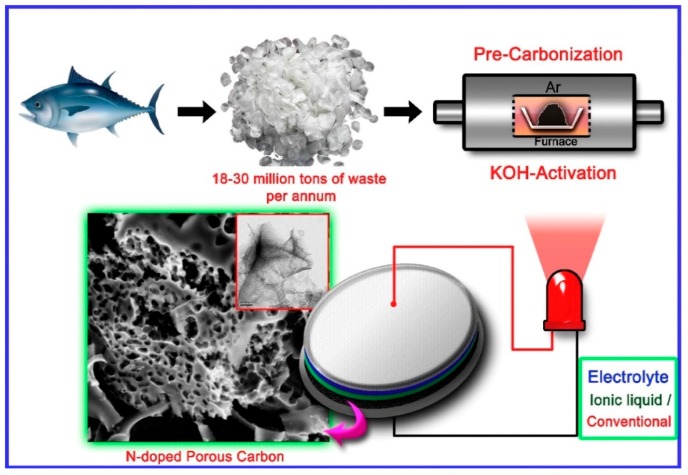
Steps of N-doped FSHC derived from fish scale and its application as an anode material for lithium ion batteries (LIBs). Reprinted with permission from Selvamani et al. [143]. Copyright 2015, Elsevier.

**Figure 12:**
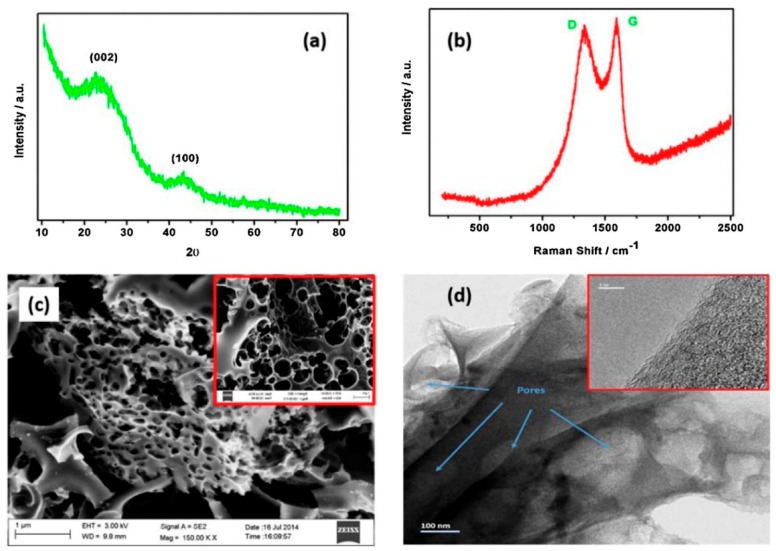
(**a**) XRD pattern; (**b**) Raman spectrum; (**c**) FE-SEM and (**d**) HR-TEM images of the N-doped FSHC. Reprinted with permission from Selvamani et al. [143]. Copyright 2015, Elsevier.

Liu et al. [150] needed a KOH chemical activator and heat treatment at 950 °C for 1 h to improve the specific surface area slightly above 2700 m^2^/g. Pyrrolic nitrogen and quaternary nitrogen dominated among nitrogen containing surface species. Zhao et al. [151] also manufactured nitrogen-rich carbon fish scales. Again, Zhao et al. applied a chemical activator, i.e., KOH (mass ratio 1:1) and calcination at 900 °C. Optionally, some sulfur was inserted by secondary heating at 155 °C for 6 h in the presence of sulfur. Non-sulfur carbons had a well-developed surface area and a total pore volume (2441 m^2^/g and 1.69 cm^3^/g, respectively). A degradation of the structural parameters was observed after embedding sulfur in the pores (surface area 229 m^2^/g). An analogous synthesis was announced by Selvamani et al. [143], who used KOH in a mass ratio 1:1 to fish scale and carbonized the mixture at 850 °C for 1 h (Figure 11). The carbons had a well-developed surface area of up to 1980 m^2^/g, and pores had a uniform size. To summarize, the nitrogen content did not exceed 3.95 at % X-ray diffraction pattern of the N-doped fish scale hierarchical carbon (FSHC) (Figure 12a) revealed that the samples were predominantly made up of randomly oriented carbon layers with pores or voids. The degree of graphitization has been determined by Raman spectroscopy (Figure 12b). The material exhibits typical D and G band at about 1330 cm^−1^ and 1590 cm^−1^, corresponding to the reflection of detect and disorder induced graphite layer (D-band), and the ideal sp^2^ configuration of the graphite sheet (G-band), respectively. The material exhibits three-dimensional hierarchical porous morphology with well-defined interconnected sheets and pores (Figure 12c,d).

Guo et al. [152] proposed a slightly different strategy of obtaining a nitrogen-doped and porous carbon catalyst via the high-temperature carbonization of fish scale biowaste. However, a different chemical activator—ZnCl_2_—was used. Wilson et al. [153] also applied ZnCl_2_ activation for the manufacture of nitrogen-doped carbon from biomass, i.e., waste dry fish (as a source of proteins). Even at a low temperature annealing at 550 °C, high surface area carbons were produced (1001 m^2^/g and 0.719 cm^3^/g value of the total pore volume). In this case, the ZnCl_2_ to dry fish weight ratio was three. However, a better micropore volume was achieved at the ratio of 1:1. The nitrogen content (12.4–5.2 wt %) decreased with an increase of the activation temperature and the ZnCl_2_ to fish powder weight ratio.

Wang et al., similarly to chitosan-derived carbons, utilized an inert hard template, i.e., SiO_2_ nanoparticles. Mesoporous nitrogen-doped carbons were derived [154]. The substrates were mixed in the mass ratio of 1:4 dried carp to SiO_2_. After drying, the mixture was heat treated at 300 °C for 1 h, and then at 900 °C for 1 h under an N_2_ atmosphere. The BET surface area of this sample was 401 m^2^/g.

## 3. Nitrogen-Doped Carbon Materials—Applications

An important change has been observed in the chemistry of carbon in the recent years: the research topics declined from general activated carbons to activated carbons with specifically directed properties. The specificity of properties, including adsorptive and catalytic ones, is largely conditioned by the presence of certain elements on the surface of the carbon, mainly in the form of the so-called heteroatom functional groups. Recently, in addition to activated carbons containing oxygen functional groups, materials that contain functional groups in the form of nitrogen–carbon bonds have been of particular interest. Both scientific and technical literature (patents) indicate a number of potential applications of activated carbons enriched with nitrogen functional groups. By the appropriate modification of precursors or prepared activated carbons, materials with significantly better adsorption and catalytic properties, and with a strictly defined acid–base or hydrophobic–hydrophilic character, can be obtained. New strategies for material fabrication are of a fundamental importance in the development of science and technology. This chapter presents areas of research and practical applications that could be interesting for the users of activated carbon enriched with nitrogen. Nitrogen-doped carbon materials are an especially significant part in the field of research on materials for the adsorption of gases, the adsorption of metal ions, or the production of electrodes for new electrode materials in different forms for energy conversion, e.g., air–metal batteries, fuel cells, or supercapacitors. The number of publications on nitrogen-containing electrodes began to rapidly increase from the year 2010. Achievements in the design of new electrode materials based on carbon with a high nitrogen content are an important contribution to the development of electrochemistry, as described in this chapter. The comparative material under discussion in this chapter is an excellent example of how natural marine resources can be processed to marine-derived materials of high applicability in areas that are far from their mature form.

### 3.1. Nitrogen-Doped Carbon for Oxygen Reduction Reaction

The oxygen reduction reaction (ORR) is an important process in many areas involving energy conversion, corrosion, or biosensors [155]. The oxygen reduction reaction at the cathode is crucial for many processes, and effective electrocatalysts are important for applications in fuel cells or metal–air batteries [156]. Platinum-based electrocatalysts are widely used in commercial fuel cells due to their relatively low overpotential and high current density [157]. However, they have disadvantages, such as a lack of resistance to intermediates, slow kinetics, and instability during prolonged use. Together with high Pt costs and limited natural reserves, these shortcomings lead to the search for alternative materials characterized by low production costs and high electrocatalytic performance. In this context, metal-free ORR-based electrocatalysts have generated considerable interest due to their low price and high activity, as well as their electrocatalytic selectivity and durability [158]. An alternative solution to commonly used platinum catalysts is the use of nitrogen-doped nanostructured carbon materials [159]. A confirmation of the application character of nitrogen-doped carbon in the ORR is the number of publications appearing over the last few years. Based on the database on the website Web of Science (keyword: nitrogen-doped carbon for oxygen reduction reaction), over 700 publications have been noted in the year 2017 alone, which confirms the topicality of this field of research.

An impulse to intensify the research on nitrogen-doped carbons was to prove that selected nitrogen functional groups can act as catalytic centers in oxygen reduction reactions and/or stabilize reactive carbon atoms and catalyze this process. The physical and chemical properties of carbon materials, such as their adaptable porosity and surface chemistry, predestine them for applications in many catalytic processes [160]. Traditional carbon materials, such as activated carbon and carbon black, can be also used as a catalytic carrier in catalytic heterogeneous processes [161]. The surface chemistry of a standard activated carbon can be easily modified, e.g., by oxidation and increase in their hydrophilicity [162] and/or insertion of other heteroatoms. In addition to the easy adaptation of the porous structure and surface chemistry, other advantages of carbon materials are presented, such as: (i) the deposition of metal ions and their reduction; (ii) the resistance of the carbon structure to acids and bases; (iii) structural stability at high temperatures; (iv) the ability to be obtained in various physical forms, such as grains, materials, fibers, granules and others; (v) the active phase can be recovered; and (vi) the costs of conventional carbon media are lower than other media. Each of these effects can be used in the synthesis of carbons in order to direct their catalytic properties towards ORR. Understanding the ORR mechanism plays a key role in the preparation of more efficient and more durable catalytic systems.

Gong et al. [163] demonstrated that nitrogen-doped carbon nanotubes as ORR catalysts display excellent oxygen catalytic performance, and are potential mechanisms for ORR on N-doped carbon materials. The literature demonstrates that three nitrogen-based species i.e., pyridinic N, pyrrolic N, and graphitic N, can enhance the ORR performances of carbonaceous materials in different ways [164,165]. Pyridinic nitrogen, which has a lone electron pair in the plane of the carbon matrix, can increase the electron donor property and facilitate reductive O_2_ adsorption [166]. Pyrrolic N can change the band structure of carbon, raising the density of π states near the Fermi level and reducing the work function, which is not an effective promoter for ORR activity, as evidenced by the sluggish activity [167]. Graphitic nitrogen atoms reduce the electron density on the adjacent C nuclei and help the electron transfer from adjacent C to N atoms. Meanwhile, N back donates electrons to adjacent Cp_z_ orbital, thus facilitating the O_2_ dissociation on the adjacent C atoms and forming a strong chemical bond between O and C [168]. Guo et al. [45] deduce that the higher onset and half-wave potentials for heteroatoms quaternary-doped carbon catalysts are simultaneously dependent on the coordination effect of S, P, and Fe-Nx species and graphitic-N, while the larger limiting diffusion current is attributable to its relatively high surface and porosity.

Mesoporous carbon materials have been widely used for ORR owing to their excellent textural characteristics, mechanical/thermal stability, and mesoporous network [169,170,171]. The properties of mesoporous carbon materials rely on not only their sizes and the size distribution of their mesoporosity, but also the heteroatoms doped into their structure [172]. Also as shown in the paper by Wang et al. [60], carp is a good precursor for the production of mesoporous N-doped carbons for ORR. The ORR activity of the obtained carbon materials containing pyridinic N groups is comparable to that of commercial Pt/C catalyst. The ORR polarization curves of N-doped and commercial Pt catalyst showed an initial sharp increase followed by a saturation effect, implying that ORR was a diffusion-controlled four-electron reduction process. Limiting current density in ORR was comparable to the performance of commercial Pt/C carbons for electrode manufacturing. Generally, the high activity towards ORR was attributed both to high pyridinic nitrogen content and mesoporous architecture.

A microbial fuel cell (MFC) is a bioelectrochemical system that drives an electric current by using bacteria and mimicking bacterial interactions found in nature, representing a novel and green technology alternative to traditional devices incorporating carbon electrodes [173]. However, several limiting factors, such as slow anodic and cathodic reaction rates, a high ohm resistance, and a high mass transfer resistance to the active sites of the electrode, hindered their widespread application. The high overpotential and sluggish reaction kinetics of the ORR are considered to be major limitations, too [174,175]. On the contrary, as shown by Fan et al. [58], the MFC with carbon obtained from *Chlorella pyrenoidosa* at 900 °C delivered the highest maximum power density of 2068 ± 30 mW/m^2^, which was about 13% higher than that of Pt/C (1826 ± 37 mW/m^2^) with the same catalyst loading. Additionally, the authors noted that the cost of the carbon received from *Chlorella pyrenoidosa* was evaluated to be ~0.25–0.37 $/g, according to the cost of the raw materials and energy consumption, which was 160-fold lower than the ~56.65 $/g of the Pt/C catalyst. Therefore, the application of *Chlorella*-derived carbons could be a green, cost-efficient alternative to Pt/C for MFC applications due to the combined effect of a high graphitization, an appropriate N and P doping, and a high ratio of the mesoporosity and macroporosity of the sample. Recently, *Chlorella* was used as a carbon precursor, but involved some new additives in synthesis: cobaltous acetate and melamine [154]. The received catalyst-containing cobalt and nitrogen exhibited an excellent electrochemical activity toward both ORR and oxygen evolution reaction (OER) in an alkaline medium. This work presents a new path to the design and development of novel types of ORR/OER bifunctional-doped carbon catalysts using abundant, low-cost, and eco-friendly natural biomass as the precursor. A different attempt was presented by Escobar et al. [51]. Metal-free carbon electrocatalysts were received from *Sargassum* spp. KOH was a chemical activator. Nitrogen content was below 2 at %, and contained mainly two types of nitrogen groups: pyridinic N and pyrrolic N.

Liu et al. [150] determined the electrochemical active surface area (ECSA) for N-doped hierarchical lamellar porous carbon synthesized from fish scales, which were applied as a support material for the Pt nanoparticle (NP) electrocatalysts. The Pt domains were uniformly dispersed on the porous surfaces of the NP electrocatalysts. Pore size distribution was very narrow, and the average pore size was ca. 2 nm. The small particle size (NP) and high dispersion of Pt significantly improved the activity toward the ORR. Compared with the Pt/Vulcan commercial carbon black, the obtained Pt/NP exhibits larger ECSA and a better catalytic activity toward the ORR (four-electron transfer pathway). The hierarchical lamellar pore structure, large surface area, and functionalized surface state are amenable to promoting the catalytic properties of Pt NPs. A low-cost production makes such materials a promising alternative to mote traditional ORR electrocatalysts.

Guo et al. [138] manufactured a three-dimensional (3D) hierarchical N-doped carbon nanoflower derived from chitosan and used as metal-free ORR electrocatalysts. These materials exhibit a high electrochemical activity comparable to that of commercial Pt/C (20 wt %), and much better durability. Also, in the paper of Liu et al. [107], 3D N-doped porous carbons were obtained, which, as electrocatalysts for ORR in alkaline media, exhibit catalytic activity with an onset potential of 0.06 V, and a high limiting current density of 5.3 mA/cm^2^ (at −0.4 V, vs. Ag/AgCl), which is comparable to that of the commercial Pt/C catalyst with an onset potential of −0.03 V.

Song et al. [63] synthesized a carbon material from *Undaria pinnatifida.* It showed excellent electrocatalytic activity in the ORR in alkaline media, which can be addressed in terms of the presence of the nitrogen and sulfur heteroatoms, as well as a well-developed porosity and electrical conductivity in the carbon framework. The pyrolysis temperature was a key controlling parameter that determined the trade-off between heteroatom doping, surface parameters, and electrical conductivity. Samples prepared at 1000 °C showed the best ORR performance. Additionally, these carbons exhibited enhanced durability and methanol tolerance relative to the state-of-the-art commercial Pt/C catalyst. Thus, that seaweed-derived carbon is a promising alternative to costly Pt/C catalysts. Another example of using carbon obtained from seaweed is a paper described by Liu et al. [57]. This catalyst exhibited improved electrocatalytic activity, long-term operation stability, and high CH_3_OH tolerance for ORR in alkaline fuel cells compared with commercial Pt/C catalyst.

Zhang et al. [109] immobilized Co/CoO nanoparticles on nitrogen-doped carbon, which was manufactured from shrimp shells. These materials were tested as electrocatalysts for ORR.

El-Negar et al. [139] obtained a hybrid catalyst (Nd_2_O_3_/N-doped carbon) derived from crustacean shells; this showed a superior ORR activity, with the highest ORR onset potential in alkaline solutions. A synergetic effect between Nd_2_O_3_ and the nitrogen surface species was noticed. The same behavior was observed in acidic media, whereas Nd_2_O_3_/N-doped carbon exhibited comparable activity to the commercial Pt/C electrode materials and much better activity compared to single Nd_2_O_3_ and N-doped carbon. ORR was assumed as a four-electron process as in the case of high-quality commercial Pt/C catalyst. Interestingly, a four-electron reduction process will reduce the amount of formed hydrogen peroxide, resulting in considerable electrode instability. In contrast, the pure Nd_2_O_3_ electrodes exhibited a lower ORR activity, with calculated electron transfer numbers of ~2.4.

### 3.2. Nitrogen-Doped Carbon for Supercapacitors and Electric Double Layer Capacitors

Carbon materials are commonly used for the fabrication of electrodes in electrochemical capacitors. Depending on the type of accumulated energy, capacitors can be divided into electric double layer capacitors (EDLC) and pseudocapacitance capacitors [176,177]. For EDLC, only electrostatic interaction occurs between the ions and the carbon surface. In the case of pseudocapacitance, the capacitance additionally uses Faraday’s redox reactions to accumulate the electric charge [34,178,179,180]. The main factors that enable choosing carbon materials as electrode materials are the following: a very well-developed surface area, appropriate pore geometry, pore size distribution (PSD), high conductivity, good wettability, and appropriate surface functional groups. The most critical seems to be a compromise between a high surface area (to ensure high capacity) and the presence of broad pores (to allow easy access to the electrolyte) [181].

Nowadays, many supercapacitors contain activated carbon as an electrode material. To increase the energy density, many researchers have tried to develop suitable carbon materials with surface functional groups of a proper nature and concentration. These groups can significantly affect the total capacity through the pseudocapacitance effects, and can improve the wettability of porous carbons by electrolytes. Recent research has shown that nitrogen-doped carbon seems to be the most promising way of increasing the electric capacity of supercapacitors while maintaining excellent durability. These properties are crucial for supercapacitors with a water-based electrolyte, and enable a multiple increase in the electric capacity in relation to EDLCs. Redox reactions on nitrogen-containing functional groups may be responsible for pseudocapacitance in water electrolytes. Non-aqueous electrolytes may have a different mechanism that uses nitrogen functional groups [177]. Due to high electric and improved hydrophilic properties, along with their easier synthesis and functionalization, nitrogen-doped carbons showed great opportunities for energy storage [182].

Before reviewing the application of marine-originated feedstock for supercapacitors electrode, making a general overview on supercapacitor electrode issue should be presented first. One of the possible ways to insert nitrogen is a gas phase ammonization. Some papers dealing with this method of nitrogen insertion proved the general importance of nitrogen surface species. Such obtained carbons were characterized as electrodes in supercapacitors by Jurewicz et al. [183]. Lota et al. [178] investigated the capacitive properties of activated carbon, and attempted to explain the role of nitrogen chemically bounded to graphene layers. The nitrogen-doped carbon materials (nitrogen content from 1.9 wt % to 7.2 wt %) were tested in supercapacitors. The beneficial effect of the presence of nitrogen on the electric capacity in acidic electrolytes was demonstrated. A series of carbons with a similar texture (S_BET_ 800 m^2^/g and more and the micropore volume of 0.84–0.93 cm^3^/g) was tested. A very good correlation of the specific capacity and nitrogen content was achieved. The nitrogen insertion triggered another phenomenon. The high concentration of hydrophilic polar sites i.e., nitrogen surface species increased in the wettability of the surface by water. Since most of the nitrogen was at the edges of the graphene layers, this effect was spectacular.

Ammonization is not a sole way to bond nitrogen to carbon matrixes applicable to supercapacitors. Typically, a synthetic precursor may be carbonized for this purpose. The major precursor were: melamine [9,184,185], polyacrylonitryle [186], polyaniline [13] derived from polypyrrole [176], aniline [187], organic salt (EDTA) [27], melamine–formaldehyde [4], and melamine–formaldehyde resin [179,188]). N-doped carbons from natural feedstock (including marine originated) are an alternative to synthetic precursors. Mesoporous nitrogen-doped carbons are favorable, as described in some papers [189,190,191].

Besides structural parameters, nitrogen content remains an important factor for a desirable electrochemical performance, as demonstrated by Hu et al. [192]. According to the announcement, nitrogen content of 6 wt % was optimal, and higher values did not bring better results. A better electrochemical performance can also relate to other additional heteroatoms (e.g., phosphorus, boron) in addition to nitrogen [193,194]. The parallel bonding of nitrogen and phosphorus to the surface increased the heterogeneity of the carbon surface as beneficial for the electrical capacity of the electrodes [193].

Several papers demonstrated that biomass-originated activated carbons offer positive features regarding their application to supercapacitors. This applies to marine feedstock as well.

Wang et al. [72] realized cyclic voltammetry experiments and determined galvanostatic charge/discharge curves for cyanobacteria originated nitrogen-doped carbons (activated by KOH). The KOH activation was inevitable to facilitate such positive changes of such parameters as specific surface area, pore structure, and degree of graphitization.

Li et al. [74] described the synthesis of nitrogen-doped nanofibers from alginate having large mesopores (~10–40 nm). These materials exhibit a large reversible capacity of 625 mAh/g at 1 A/g, and excellent cyclic performance. To investigate the stability of the nitrogen-doped nanofibers obtained at 600 °C, the galvanostatic charge/discharge cycling tests were performed at a current density of 5 A/g in 5000 cycles. This material can have 91.7% of the initial specific capacity after 5000 cycles, indicating its good cycling stability for supercapacitors.

Despite the origin of nitrogen-doped carbon some effects are crucial all these materials including marine feedstock derived. A high contribution of mesopores to the total pore volume facilitates short ion-transport pathways and low inner-pore resistance [195,196]. The N-doping enhances the electrical conductivity [191,197,198]. Pyridinic and pyrrolic-type N existing on the carbon surface can act as the electron donor and/or enhance the chemisorption of the space charge layer for promoting the chemisorption of the electrolyte ions [199,200,201].

These general remarks are reflected in a paper that focused on the marine/freshwater feedstock-based manufacturing of electrode carbons, as described by Lee et al. [70,71]. Nitrogen-doped carbons were obtained from *Chlorella vulgaris* by KOH activation. The activated carbon electrodes for EDLCs had a high specific capacitance of approximately 117 F/g at 0.5 A/g, and had better electrode properties compared with commercial activated carbon. However, when the nickel oxide was inserted into the N-doped activated carbons, the composite had the highest capacity of 624.2 F/g at 1 A/g. After the addition of a nickel derivative, the specific capacitance increased by 530% compared to the commercial activated carbon. The composite electrode exhibited good retention of the specific capacitance for 1000 cycle tests.

Chen et al. [145] examined a fish scale-based hierarchical lamellar porous carbon, and its electrochemical performance as an electrode material for EDLC. The cyclic voltammetry (CV) curves exhibit an ideal capacitor property by showing a typical rectangular CV curve without any redox peak in the chosen voltage range.

Shen et al. [69] described the synthesis of an activated carbon from phytoplankton and KOH as the activator. Such carbon-based supercapacitors had a high capacitance of 130 mF/cm^2^ at the current density of 2 mA/cm^2^, and good stability (92% capacitance retention after 10,000 cycles).

Hao et al. [142] synthesized N-doped carbon from natural sepia ink. It was tested as an EDLCs electrode, and exhibited a remarkable specific capacitance of 320 F/g at the current density of 0.5 A/g. Furthermore, the assembled EDLCs demonstrated a good energy density of 28 Wh/kg and a power density of 625 W/kg (86% capacitance retention after 10,000 cycles within the voltage window of 0–2.5 V in the organic electrolyte).

Yu et al. [56] obtained nitrogen and oxygen co-doped hierarchical porous carbons from algae—*Enteromorpha* and applied them in ionic liquid (electrolyte) supercapacitors of superior features: specific capacitance of 201 F/g at 1 A/g and 20 °C, a capacitance retention ratio of 61 at % 100 A/g, and a capacitance loss of 9% after 10,000 cycles. The devices were able to deliver an energy density of 24 or 35 Wh/kg at an extremely high power density of 60 kW/kg at 20 or 60 °C.

To sum up, the presented review of the papers allows us to assume that, in the case of applications in supercapacitors, nitrogen functional groups have positively influenced the increase in pseudocapacitance. In addition, electrolyte access to the active surface is improved by improving the wettability of the electrodes. It was shown that the influence of the four most dominant nitrogen forms, i.e., pyrrole, pyridine, quaternary/graphite, and pyridine N-oxides, which have a large share in the generation of pseudocapacity, specific capacity, and ensure stability of properties, are particularly significant.

### 3.3. Nitrogen-Doped Carbon for Batteries

Rechargeable lithium ion batteries (LIBs) are considered effective and modern devices for energy storage [202,203,204]. Among the various components of the LIBs, carbonaceous materials have been studied extensively as potential anodes because of their easy availability, high charge capability, and appreciable cycling resistance [205,206]. Besides that, heteroatom (e.g., P, N, S, and B) doping can also provide many active sites to improve Li^+^ storage capacity [207,208,209].

Generally, the nitrogen atoms own a lone pair of electrons, and the electronegativity of nitrogen is higher than that of carbon, which can break the electroneutrality of the carbon surface to create the active sites. Additionally, the N-doping sites are more favorable to bind with Li^+^, resulting in an increased electrochemical performance.

Many papers report different strategies in the production of N-doped carbons and their applications as an anode material with a high capacity and low cost, which is also environmentally safe for different types of batteries. Yu et al. investigated N and O co-doped hierarchical porous carbons derived from algae. The carbons had a high surface area, a combined macro/meso/micro pore structure, and were used as electrodes in LIBs [56]. The results showed that the anode (in the LiPF_6_ electrolyte) exhibited a high specific capacity of 1347–1709 mAh/g, and a good cyclability up to 500 cycles. The authors believe that this simple precursor synthesis way offers excellent potential for facile large-scale material production for lithium ion batteries. Liu et al. [75] investigated a seaweed-derived Fe_2_O_3_ nanoparticles/N-doped graphene aerogels as an electrode material for LIBs. Remarkable cyclic stability (729 mAh/g at 0.1 A/g for 300 cycles) was achieved, outperforming all of the reported Fe_2_O_3_/graphene hybrid electrodes.

Kovalenko et al. [53] prepared carbons from brown algae for LIBs. Li et al. [74] obtained hierarchical carbon nanofibers (CNFs), providing a three-dimensional pathway for ion transport. A series of high-performance electrodes for LIBs was manufactured and tested. They exhibited a large reversible capacity of 625 mAh/g and superior stability. The importance of ion and electron transport in CNFs was underlined in other papers as well [210,211]. Nowak and Lisowska-Olesiak [77] obtained carbon materials from red algae, and tested them as anodes for lithium batteries. The best results for all of the tested samples were observed when the charging/discharging process was slow. The highest capacity value was observed for a sample obtained with an additional component i.e., 10% of glucose (633 mAh/g).

**Figure 13:**
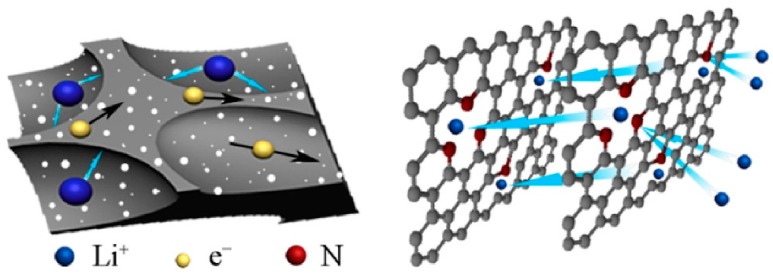
Schematic illustration of advantages of N-ACAs in enhancing the performance of LIBs. Reprinted with permission from Zhang et al. [68]. Copyright 2017, Elsevier.

Zhang et al. [68] derived macroporous and mesoporous N-doped carbon aerogels (N-ACAs) from seaweed and tested them an anode material (Figure 13).

#### 3.3.1. Lithium-Sulfur Batteries

Hierarchical porous carbon has been successfully employed for encapsulating sulfur in the assembly of high-performance lithium–sulfur (Li–S) batteries [212,213,214]. Li–S batteries, with their high theoretical specific capacity (1672 mAh/g) and theoretical specific energy density (2600 Wh/kg), are a promising alternative to current generation Li ion batteries that are approaching their theoretical capacity, but are insufficient for large-scale energy storage and long-range electronic vehicles [215]. In the past decade, although high-cost carbon nanotubes, carbon nanofibers, and graphene have been intensively studied to overcome the battery shortcomings, only limited progress has been achieved [213,216,217]. Therefore, hierarchical porous carbon fabricated from biomass (especially marine-derived feedstock) has received increased attention, due to the desirable properties of Li–S cells [218,219]. The seaweed derived N-doped carbons may have well developed micropores, mesopores, and macropores, which were beneficial for encapsulating a large amount of sulfur [61]. The such obtained sulfur–carbon composite contained 65.7 wt % of sulfur, had a high initial discharge capacity of 1221.2 mAh/g, and retained a capacity of 826.4 mAh/g after 70 cycles at 0.2 C. Additionally, the composite retained a reversible capacity of 540.6 mAh/g after 300 cycles at a high rate of 1 C. Compared to the pure sulfur cathode, the composite cathode displays excellent rate capabilities, low polarization, and good reaction kinetics, highlighting that biomass-derived porous carbon is suitable for assembling high-performance Li–S batteries. Zhao et al. [66] synthesized hierarchical porous carbons derived from green algae, which enable the fabrication of a sulfur composite that was applicable as a cathode material for high performance Li–S batteries. The good electrochemical performance of the composite could be attributed to a unique combination of mesoporosity and surface chemistry, allowing the retention of the intermediate polysulfides within the carbon framework. The natural nitrogen doping of carbon can obviously improve the electron conductivity and strongly adsorb polysulfides, which will result in a good rate capability and remarkable cycling stability for Li–S batteries. A porous sulfur/carbon composite was obtained from fish scales and applied in Li–S batteries by Zhao et al. [151]. The porous nanostructure of the composite is beneficial for enhancing the life cycle of Li–S batteries by adsorbing the polysulfide produced during the electrochemical reaction. The resulting nanocomposite shows a high capacity of 1039 mAh/g at 1 C (1 C = 1675 mA/g) in the first cycle, and the reversible capacity remains high up to 1023 mAh/g even after 70 cycles.

Another 3D hierarchical nitrogen-doped carbon (NCNF) derived from chitosan was used as the electrode material for Li–S batteries, as described by Guo et al. [138]. NCNF had a high loading content of sulfur (80 wt %), which enabled a high capacity of 1633 mAh/g, a remarkable rate capability of 916 mAh/g, and good cycling performance, with a capacity decay of 0.07% per cycle over 500 cycles.

#### 3.3.2. Lithium-O_2_ Batteries

The rechargeable Li–O_2_ battery has recently attracted a great deal of attention because of its significantly higher theoretical energy density than the traditional lithium ion battery [220,221]. A Li-O_2_ battery prototype is composed of a lithium metal anode, a Li^+^-containing non-aqueous electrolyte, and a porous O_2_ cathode. Zang et al. [108] elaborated a binder-free N-doped carbon paper electrode (NCPE) of a “bird’s nest” microstructure, which could provide mechanic durability, fast O_2_ diffusion, and enough space for discharge product deposition. The N-doped carbon in the NCPE provided the N-containing function groups, which can promote the electrochemical reactions. The first discharge capacity for a Li–O_2_ cell with the NCPE at a current density of 0.1 mA/cm^2^ reached 8040 mAh/g, with a cell voltage of around 2.81 V. In addition, the cell with the NCPE presented a cyclability of more than 30 cycles at a contrast current density of 0.1 mA/cm^2^. It exhibited a coulombic efficiency of 81% on the first cycle at a current density of 0.2 mA/cm^2^.

### 3.4. Nitrogen-Doped Carbon for Adsorption

Activated carbons are widely used in many areas of everyday life and numerous industrial and environmental processes. Examples include the purification and separation of gases as well as solvent regeneration or wastewater treatment. Depending on the used starting material (precursor), carbons of various structural, mechanical, and adsorption properties are obtained. From the literature data, it can be concluded that activated carbons modified with nitrogen may play a very important role in adsorptive applications. This fact is confirmed by the number of publications, which has been growing significantly over the last six years. Generally, activated carbons with a high nitrogen content are described in the literature in the two contexts of their synthesis, which is a certain technological challenge, as well as specific properties for practical applications. In the last few years, research studies were focused on the use of nitrogen-containing carbons as specific adsorbents with considerable surface alkalinity due to the presence of an active pair of electrons on surface nitrogen atoms in a series of reactions. In particular, they are the adsorption of acidic oxides in the gas phase and the binding (complexation) of transition metal cations in the liquid phase. In this approach, carbon materials containing nitrogen are used in the widely-understood environmental protection as well as the protection of human health and life. In environmental protection, they can be used as effective adsorbents of heavy metals from aqueous solutions and as adsorbents of these acidic oxides.

The literature shows that nitrogen-doped carbons applied as selective adsorbents are mostly derived from non-marine feedstock, in which application for such carbon manufacturing is accidental (only a few examples are given). Nevertheless, such an application will be reviewed on the example of nitrogen-doped carbons obtained from other feedstock just to demonstrate the basic requirements and the openness of this specific application field (selective adsorption of gas and liquid phase). In summary, the application of marine/freshwater-derived feedstock is still a non-discovered field of science and technology. Marine/freshwater-derived nitrogen-doped activated carbons may be produced in mass quantities due to high accessibility and the low cost of the feedstock. Such feedstock features does not apply to some synthetic precursors such as polyaniline, which in fact are niche from a mass fabrication point of view. Mass quantities of nitrogen-doped carbon adsorbents are definitely required by air and water purification, CO_2_ sequestration, etc. Extensive activity is still required in the form of industrial research and experimental development aiming at the implementation of marine/freshwater-derived carbon adsorbents to purification purposes. An extensive literature and patent search revealed the lack of a solution towards the adsorption of biologically/therapeutically active molecules on nitrogen-doped carbon adsorbents, in particular the ones obtained from marine feedstock. The research space is still waiting for new contributions. Especially, drug accumulation and controlled delivery is a blank gap, which is rather astonishing regarding the specific adsorption properties of the carbon. Surprisingly, drug accumulation/delivery by means of less specific standard carbon materials is widely recognized by the scientific audience [222].

#### 3.4.1. Removal of Pollutants from the Gas Phase

As has been shown in the current research, the presence of nitrogen functional groups in the carbon matrix improves the ability to capture acidic gases, such as SO_2_, SO_3_, or CO_2_. These properties are different, and are often dependent on the nature of the nitrogen groups in the research material. Chen et al. used nitrogen enriched carbon nanofibers in SO_2_ and CO_2_ adsorption [223]. Cyclic adsorption/desorption experiments confirmed a high stability of the experimented fibers for over 20 SO_2_ experiments and 15 CO_2_ experiments. These materials also showed favorable properties in the Cd^2+^ adsorption. In another paper [224], the authors demonstrated that the adsorption SO_2_ was dependent on the size of the pores and the amount of nitrogen functional groups incorporated in the carbon structure. Raymundo et al. [225] investigated the removal of SO_2_ from the flue gas with nitrogen-doped activated carbon (nitrogen content below 5 wt %) in the form of powder and fibers. The materials were also active as an oxidative catalyst: SO_2_ to SO_3_. Boudou et al. [226] investigated carbons obtained from men viscose cloths activated with ammonia as catalysts for the oxidation of H_2_S and SO_2_. The obtained carbon materials were characterized by low surface areas (S_BET_ <30 m^2^/g). The adsorption of some acidic oxides was investigated by Bashkowa et al. [227]. The authors described the catalytic oxidation of hydrogen sulfide on activated carbon impregnated with urea before carbonization. The highest capacity and selectivity were found for nitrogen-containing carbons in the amount of 1–1.5% by weight. The obtained results, similar to other papers, confirmed that the selectivity of the activated carbon used as a catalyst in the process of H_2_S oxidation depended on the textual, surface, and structural chemical properties of the carbon surface. Similar conclusion were presented in another paper [228]. The oxidation of H_2_S proceeds through a stepwise reaction in the pores (from smaller to larger), with the participation of HS^-^ ions. The following factors played a special role: (1) the presence of small micropores; (2) a high total volume of micropores; and (3) the presence of dispersed nitrogen base groups embedded in the carbon matrix.

A further example of applications in the acid gas adsorption is the paper of Fan et al. [126], the aim of which was to effectively adsorb CO_2_. The authors describe the effect of nitrogen functional groups in porous carbon regarding its adsorptive capacity towards CO_2_. Similar studies were carried out by Li et al. [229] and Thote et al. [31] (a carbon obtained from soybeans and ZnCl_2_ activator). The high adsorptive capacity towards CO_2_ was attributed to the presence of pyridine groups. Bagreev et al. [230] showed that the impregnation of carbons with melamine (heated later at 850 °C) caused a significant increase in the nitrogen content, which contributed to increasing the capacity of these materials in the effective removal of methyl mercaptan. Another adsorption-oriented application was presented by Abe et al. [231], who examined the adsorptive properties of activated carbons with amine groups (nitrogen content of 1.6–1.8 wt %). The conducted research evidenced that the introduction of amino groups had an extremely favorable effect on the adsorption of NO from the gas phase. The effectiveness the removal of nitrogen oxides is also described by Beshkova et al. [232]. The authors focused their research on the use of carbon obtained from wood modified with urea in the process of nitrogen(IV) oxide removal. In particular, carbons obtained at 950 °C showed a positive effect on NO_2_ adsorption and NO retention. As indicated by the authors, it is possible that the presence of nitrogen groups on the surface of carbon and some increase in the volume of the supermicropores played a key role in the process of removing NO_2_. In the paper of Burg et al. [233], nitrogen-containing carbon was obtained from lignite and urea (1:1 ratio; the reaction was carried out in an autoclave at 350 °C) and then activated with steam. The obtained material showed adsorption properties towards volatile organic compounds (VOC). It was observed that the enrichment of the material with nitrogen (1.8–3.8 wt %) contributed to the increase in the activity in VOC adsorption.

Carbon materials have been extensively studied as candidates of hydrogen storage, owing to their large surface area, well-developed pore structure, low mass density, and low cost [234,235]. Heteroatom doping is widely used to modify carbon materials for efficient adsorption. For the adsorption of hydrogen, Li et al. investigated the influence of doping with nitrogen, sulfur, and phosphorous on the adsorption of H_2_, CH_4_, and CO_2_, which exhibited a favorable effect on the adsorption of H_2_ [236]. Giraudet et al. performed mesoporous carbons synthesized using ammonia during thermal processing [237]. As a result of the applied procedure, materials with a nitrogen (from 3.9 wt % to 19.6 wt %) and some content of nickel were obtained. The hydrogen storage capacity at temperatures above 25 °C was increased due to the presence of both nitrogen and nickel doping. In general, micropore volume is essential for enhancing the uptake of hydrogen [238,239]. Guo et al. [73] researched the effect of nitrogen and phosphorus heteroatoms in cyanobacteria-derived activated carbon for H_2_ adsorption. In comparison with the surface groups, the S_BET_ should have a much greater effect on the uptake of H_2_.

#### 3.4.2. Removal of Pollutants from the Liquid Phase

Another area of application for carbons containing nitrogen functional groups is adsorption from the liquid phase. Among them, important parameters are: the physicochemical nature of adsorbents, the nature of the adsorbate, and the process environment. In the light of the conducted research, it can be assumed that the chemical nature of the surface and the porous structure are the most important factors affecting the sorptive properties in the liquid phase. An example of this type of applications is the Cu^2+^ adsorption on activated carbons that are preoxidized in HNO_3_ and preheated in an ammonia atmosphere, as described by Biniak et al. [240]. Adsorption of the copper cation was investigated by means of spectroscopic and electrochemical methods. It has been shown that the number of adsorbed ions depends on the acid–base nature of the surface, as well as on the pH of the solution. Among the possible interactions between the metal ion and the surface of activated carbon, the formation of complexes with nitrogen or/and oxygen groups can be mentioned. Another paper [241] describes the adsorption of heavy metal ions (Cd^2+^, Pb^2+^, Cu^2+^, Hg^2+^) on powdered activated carbon materials. The samples of heat-treated carbon were altered by heating in an atmosphere containing oxygen and ammonia and oxidized with concentrated HNO_3_.

Xiao et al. researched two series of activated carbons containing oxygen and nitrogen functional groups [242]. One series with a low nitrogen content (~1 wt %) was obtained from coconut shells. The second one with a high nitrogen content (~8 wt %) was obtained from polyacrylonitrile. For both series, the connection between the type and content of the functional groups was examined, without determining the effects of the different porous structures. The maximum amount adsorbed in the oxidized activated carbons determined by the Langmuir equation followed the direction Hg^2+^ (aq) > Cu^2+^ (aq) > Pb^2+^ (aq) > Cd^2+^ (aq) > Ca^2+^ (aq). The number of adsorbed Cd^2+^ (aq), Pb^2+^ (aq) and Hg^2+^ (aq) ions decreased with a decreasing concentration of surface oxygen functions. Adsorption on nitrogen functional groups was related to the coordination mechanism. The changes in the sorption capacity range were related to the decomposition of surface carboxyl functional groups and the occurrence of pyridine functional groups.

Other research [243] confirmed the applicability of nitrogen-containing carbons for the adsorption of aromatic compounds, such as nitrobenzene, benzoic acid, phenol, or aniline. The tested adsorbents were activated carbons containing from 1.6 at % to 4.2 at % nitrogen. Yu et al. explored the impact of acid treatments on the N-doped porous carbon obtained from fish scales and its Cr(VI) removal capability [148]. It was found that adsorbed Cr(VI) ions were partly reduced in the Cr(III) species. The as-produced Cr(III) was in proportion to the content of quaternary nitrogen in the carbons.

### 3.5. Biomedical Application of Nitrogen-Doped Carbon Materials Obtained from Marine Resources: Bioimaging

Until now, the biomedical applications of nitrogen-doped carbon materials were investigated in only a few papers. However, the results published so far show the biomedical potential of such materials. Godavarthi et al. synthetized nitrogen-doped carbon dots (NCDs) from a waste seaweed *Sargassum fluitans* by a hydrothermal method [62]. The synthesized NCDs were water soluble and had a single nanometer size between 2–8 nm. Additionally, NCDs exhibited excellent fluorescent properties with a quantum yield of 18.2%. NCDs were tested as fluorophores for double-stranded DNA, single-stranded DNA, and RNA detection. The authors found that NCDs had fluorescent tagging abilities: a significant increase in fluorescence was observed upon the tagging of NCDs with nucleic acids, which enabled a very good visualization of the acids. Thus, NCDs were considered a competitive fluorophore to commercial toxic organic staining agents.

Zhang et al. proposed to reuse Nannochloropsis biocrude oil (NBO) for the synthesis of nitrogen and sulfur co-doped carbon dots (N-S-C-dots). NBO is a product of marine algae [64]. The N-S-C-dots were capable of passing through the heavily thickened wall of mature *Arabidopsis thaliana* (*A. thaliana*) guard cells. N-S-C-dots exhibited intensive multicolor luminescence, excellent biocompatibility, and a high solubility in water. The dots were applied to the bioimaging of root tissues. The study emphasized the potential application of carbon dots (CDs) for bioimaging, and demonstrated the significance of investigating the reuse of marine algal biomass.

## 4. Summary

This paper provides a review of precursors for obtaining nitrogen-containing carbon materials with a controlled porous structure. Synthetic and non-marine feedstock, in contrast to marine and freshwater resources, suffers from several shortcomings such as insufficient nitrogen content, high price, limited accessibility, etc. It was shown that the type of marine-derived raw material, the conditions of carbonization and activation, as well as the type of activating agent make it possible to obtain a carbon adsorbents with a tailored pore distribution suited to potential applications. The review of obtained nitrogen-rich carbon materials allowed determining the direction of further research on materials for electrochemical and adsorption/catalytic applications. It seems that such research should primarily focus on planning effective manufacturing from carefully selected precursors especially those denoted as “marine-derived feedstock”. In the future, the subject of N-containing carbons will definitely decline towards the application of marine feedstock for manufacturing nitrogen-doped activated carbons, since marine resources are renewable and inexpensive.

## Figures and Tables

**Table 1:** Exemplary values of the surface area (S_BET_) and the content of nitrogen (N) in some N-rich carbons obtained from synthetic polymers.

Synthetic Polymer	S_BET_ (m^2^/g)	C_N%_	References
Melamine–formaldehyde resin	1460	7.0 wt %	[4]
Melamine–formaldehyde resin	12–56	30.3–31.7 wt %	[5]
Urea–formaldehyde resin	5–2479	3.1–12.3 wt %	[5]
Melamine resin	900	1.7–2.4 wt %	[6]
Melamine resin	1–496	3.03–4.04 at %	[7]
Polyurethane	13–112	0.71–6.31 wt %	[8]
Polyurethane	<1	7–10 wt %	[9]
Polyvinylpyridine	519–1420	1.4–2.6 wt %	[10]
Polyamide	1–1329	4.18–8.02 wt %	[11]
Polyimide	137	3.67 at %	[12]
Polyaniline	325–514	6.7–10.89 at %	[13]
Polyaniline	115–670	6.5–12.6 wt %	[14]
Polyaniline	24–94	8.7–13.3 wt %	[15]
Polyaniline	n.d. ^1^	10.3–14.9 wt %	[16]
Polyaniline	200–205	2.1–10.8 wt %	[17]
Pyrrole	900–1200	5.8–9.9 at %	[18]
Polypyrrole	2086	3.05 at %	[19]
Polypyrrole	781–1243	4.31–7.26 wt %	[20]
Polypyrrole	1060–1170	4.24–4.72 wt %	[21]
Polypyrrole	1560	5.5 at %	[22]
Acetonitrile	286–1034	7.0–8.8 wt %	[23]
Polyacetonitrile	620–832	1.7–7.2 wt %	[10]
Polyacetonitrile	544	5.3 at %	[24]
Polyacetonitrile	644–800	C/N 5.75–22.26 at %	[25]
Polyacetonitrile	520–840	n.d.	[26]

^1^ n.d.—not detected.

**Table 2:** Key stages of the first ever method for obtaining nitrogen-rich carbon materials from chitosan (method A) [112].

Stage	Agent	Aim	Result
1.	Chitosan powder and water premixed mechanically	Softening of chitosan and its transformations into a homogenous paste	Chitosan–water viscose paste
2.	Addition of aqueous HCl solution with a pH of about 7	Protonation of –NH_2_ to –NH_3_^+^ groups and testing of hydrophobic properties	Absorption of water and the formation of a low-viscosity homogeneous paste
3.	Optional addition of an aqueous urea solutionFor alternative treatments B–F, see Table 3	Optional introduction of additional amounts of nitrogenFor alternative aims B–F, see Table 3	Increased amount of nitrogen in the carbon matrixFor alternative results B–F, see Table 3
4.	Addition of an aqueous solution of Na_2_CO_3_	Introducing a substance capable to form nanocrystallites of a pore-genic hard template	Chitosan gel supplemented with a pore-genic (template) substance (Na_2_CO_3_) uniformly distributed in the gel
5.	Drying	Removal of excess water and crystallization of Na_2_CO_3_ nanocrystallites (pore-genic template) in the dry chitosan mass	Removal of excess water and crystallization of Na_2_CO_3_ nanocrystallites (pore-genic template) in the dry chitosan mass
6.	Oxygen-free carbonization	Obtaining a carbon matrix with embedded Na_2_CO_3_ nanocrystallites (pore-genic hard template)	Non-porous carbon matrix enriched with nitrogen
7.	Etching with concentrated HCl	Removal of pore-genic/template Na_2_CO_3_ nanocrystals from carbon matrix	Raw porous carbon matrix
8.	Washing with distilled water	Removal of water-soluble pollutants and Na_2_CO_3_ residues	Carbon matrix with a developed specific surface area, pore structure and a significant nitrogen content without pollutants and template residues

**Table 3:** Possible modifications of method A for the production of nitrogen-rich carbon materials from chitosan.

Method	Description of the Method	References
B	Forgo on Na_2_CO_3_ solution. Addition of an aqueous solution of ZnCl_2_. Then, steps 7 and 8 are combined and rely on long-term rinsing with hot distilled water until the chloride ions disappear.	[120]
C	Forgo on Na_2_CO_3_ solution. Addition of aqueous H_3_PO_4_ solution. Then, steps 7 and 8 are combined and rely on long-term rinsing with hot distilled water until the phosphate ions have disappeared.	[102]
D	Forgo on Na_2_CO_3_ solution. Addition of a solid and insoluble template, e.g., CaCO_3_.	[121]
U	In all variants of the synthesis method, i.e., A, B, C, or D, the addition of a water-soluble, nitrogen-containing, low molecular weight substance to increase the nitrogen content is optionally used.	[122]
E	In all variants of the synthesis method, i.e., A, B, C, D, or U, the addition of soluble metal salts is used to give new properties, e.g., catalytic or biocidal properties.	[123,124]

**Table 4:** Correlation between the synthesis method (from A to F) and selected physicochemical properties of nitrogen-rich activated carbons (as published by Ilnicka and Lukaszewicz).

Precursor	Modification/T (°C)/t (h)	S_BET_ (m^2^/g)	N (wt %)	V_t_ (cm^3^/g)	V_me_ (cm^3^/g)	References
CH ^1^ + Na_2_CO_3_	A/600/1	10–441	n.d.^2^	n.d.	n.d.	[112]
CH + ZnCl_2_	B/600–800/1	583–1932	4.5–7.5	0.31–1.33	0.008–0.507	[120]
CH + Na_2_CO_3_	A/600–900/1	441–1148	2.8–6.5	0.18–0.70	>1%	[122]
CH + Na_2_CO_3_ + CH_4_N_2_O	A + U/600/1	121–430	9.4–13.1	0.11–0.21	>1%	[122]
CH + H_3_PO_4_	C/600/1	970–1484	4.7–6.1	0.439–1.543	0.408–1.515	[102]
CH + ZnCl_2_ + Cu(NO_3_)_2_	B + E	102–1159	5.0–7.8	n.d.	n.d.	[123]
CH + Cu(NO_3_)_2_	Without template/700/1	102–123	7.8–7.9	n.d.	n.d.	[124]

^1^ CH stands for chitosan; ^2^ n.d. stands for not determined.

**Table 5:** Recent directions in the synthesis of high-nitrogen content carbon materials by means of chitosan (CH).

Year	Precursor	Synthesis: T (°C)/t (h)	Obtained Carbon Material	Applications	References
2013	CH + K_2_CO_3_	600–800/1	Microporous S_BET_ = 1.4–2469 m^2^/g, C_N%_ = 1.29–7.60 wt %	Accumulation of CO_2_	[126]
2013	CH + secondary activation of NaOH	400–600/1 later calcination with NaOH/400–600/1	Microporous S_BET_ = do 3500 m^2^/g, C_N%_ = do 5.4 wt %	Supercapacitor	[127]
2013	CH + SiO_2_	900/4	Mesoporous S_BET_ = 608–1337 m^2^/g, C_N%_ = 2.17–8.83 wt %	n.d. ^2^	[128]
2014	CH + ZnCl_2_ or KOH or CO_2_	600–850/3–5	Mesoporous S_BET_ = 912–1770 m^2^/g, C_N%_ = 3.92–5.37 wt %	Electrode material	[129]
2014	CH + SWNT ^1^	600/2	Mesoporous, conductive composite S_BET_ = 628 m^2^/g, C_N%_ = n.d.	Supercapacitor	[130]
2015	CH + FeCl_3_	450/1	Nonporous S_BET_ = 4.7–62.8 m^2^/g, C_N%_ = n.d.	Removal of Cu^2+^ ions from water	[131]
2015	CH + H_3_BO_3_	800/1	Micro/mesoporous, conductive carbon matrix S_BET_ = 3–710 m^2^/g, C_N%_ = 8.19 wt %	Supercapacitor	[132]
2015	CH, secondary activation of KOH	800/3 later calcination with KOH/700–1000/2	Micro/mesoporous containing graphene nanostructures S_BET_ = do 2435 m^2^/g, C_N%_ = n.d.	Supercapacitor	[133]
2015	CH + secondary activation of KOH	700–1000/30 min, later calcination with KOH/700–800/1	Microporous S_BET_ = 922–3066 m^2^/g, C_N%_ = n.d.	Accumulation of hydrogen	[134]
2015	Shrimp shell + H_3_PO_4_	400–600/1	Mesoporous S_BET_ = 38–774 m^2^/g, C_N%_ = 2.9–3.9 wt %	Supercapacitor	[135]
2016	CH, secondary activation of KOH	650/1 later calcination with KOH/750–850/	Mesoporous S_BET_ = 2397–2807 m^2^/g, C_N%_ = 0.2–0.4 Carbonised with CH S_BET_ = 1.3 m^2^/g, C_N%_ = n.d.	Supercapacitor	[136]
2016	CH + activation of CO_2_	900/15 min, later activation CO_2_/900/n.d.	Microporous S_BET_ = 1101 m^2^/g, C_N%_ = do 5.4 wt %	Supercapacitor	[137]
2017	CH + SiO_2_	155/12	Mesoporous S_BET_ = 907 m^2^/g, C_N%_ = n.d.	Electrode material	[138]
2018	CH + Nd_2_O_3_	500–900/2	Mesoporous S_BET_ = n.d., C_N%_ = 7.41 at %	Energy storage	[139]

^1^ SWCNT-single-walled carbon nanotube; ^2^ n.d. stands for not determined.
